# Individual and cumulative effects of social determinants of health on cardiovascular disease: Gender-specific insights from a cross-sectional NHANES study

**DOI:** 10.1371/journal.pone.0344108

**Published:** 2026-03-26

**Authors:** Xiuming Yang, Jiahui Zhou, Feier Wu, Zehu Xue, Zongliang Yu

**Affiliations:** 1 Department of Cardiology, Affiliated Kunshan Hospital of Jiangsu University, Kunshan, Jiangsu, China; 2 Department of Cardiology, Gusu School, Nanjing Medical University, The First People’s Hospital of Kunshan, Kunshan, Jiangsu, China; 3 Department of Nephrology, Affiliated Kunshan Hospital of Jiangsu University, Kunshan, China; Instituto Nacional de Cardiologia Ignacio Chavez, MEXICO

## Abstract

**Objective:**

This study aimed to examine the associations of individual and cumulative social determinants of health (SDoH) with cardiovascular disease (CVD) prevalence and sex-specific disparities among U.S. adults.

**Methods:**

Employing a cross-sectional design, we analyzed data from the nationally representative National Health and Nutrition Examination Survey (NHANES, 2005–2018). Five core SDoH domains were operationalized through eight validated sub-indicators. Associations between individual and cumulative SDoH and CVD prevalence were assessed using survey-weighted multivariate logistic regression, with sex-stratified analyses.

**Results:**

In this cross-sectional sample of 35,781 participants, adverse individual SDoH and higher cumulative adverse SDoH were associated with higher odds of prevalent CVD. In the fully adjusted model (Model 2), unemployment showed a large association with prevalent CVD (AOR = 2.27, 95% CI: 2.01–2.57). In sex-stratified analyses, point estimates for some SDoH indicators were higher in women than in men, but 95% confidence intervals overlapped for many comparisons and sex-by-SDoH interaction tests were not statistically significant (all P for interaction > 0.05). Among individual SDoH indicators, unemployment and low income (PIR < 300%), as well as food insecurity, showed the strongest independent associations with prevalent CVD.

**Conclusion:**

Both individual and cumulative SDoH were independently associated with prevalent CVD. Sex-stratified analyses suggested that some point estimates were larger in women than in men, but sex-by-SDoH interaction tests were not statistically significant.

## 1. Introduction

Globally, cardiovascular disease (CVD) is a leading cause of mortality [1]. Recent epidemiological statistics from the American Heart Association (AHA) indicate that global CVD deaths increased from 12.4 million in 1990 to 19.8 million in 2022 [2]. Projections from the Global Burden of Disease (GBD) study suggest that CVD will remain a leading contributor to global age-standardized mortality from 2022 to 2050 [3]. Social determinants of health (SDoH) refer to structural conditions that shape health trajectories across the life course, from prenatal development through older adulthood [4]. The Healthy People 2030 framework categorizes SDoH into five domains: (1) Economic Stability, (2) Education Access and Quality, (3) Health Care Access and Quality, (4) Neighborhood and Built Environment, and (5) Social and Community Context [5]. Within this framework, economic stability includes employment and income/poverty, as well as material hardship (e.g., food insecurity and housing instability). Empirical studies have linked adverse SDoH profiles to higher CVD risk and chronic low-grade inflammation [6]. CVD prevalence shows marked socioeconomic gradients, with a disproportionately higher burden among low-income groups, individuals with lower educational attainment, and residents of deprived neighborhoods, underscoring the impact of SDoH [7]. SDoH are key structural factors that mediate racial inequalities in all-cause and CVD-specific mortality by shaping access to health-promoting resources [8]. Although existing evidence underscores the central role of SDoH in population-level CVD prevention, most epidemiological studies evaluate single SDoH domains and overlook the synergistic effects of cumulative socioeconomic exposures [9,10]. Further investigation is needed to clarify how individual and cumulative SDoH are associated with CVD incidence. Given the sociodemographic complexity of the United States (US) population, population-representative and methodologically rigorous studies are needed. In this cross-sectional study, we used nationally representative data from the National Health and Nutrition Examination Survey (NHANES) to examine associations between individual-level and cumulative SDoH and CVD prevalence among adults in the US.

We hypothesized that adverse SDoH are associated with CVD prevalence in additive and potentially synergistic ways, such that greater cumulative exposure is associated with a disproportionately higher CVD prevalence. We further examined sex as an exploratory effect modifier to assess whether these associations differ between men and women. Accordingly, we aimed to (1) evaluate associations between individual and cumulative SDoH and CVD prevalence, (2) assess whether cumulative SDoH exhibit additive versus potentially synergistic patterns, and (3) explore sex-specific differences in these associations.

## 2. Materials and methods

### 2.1 Data source

**Fig 1:**
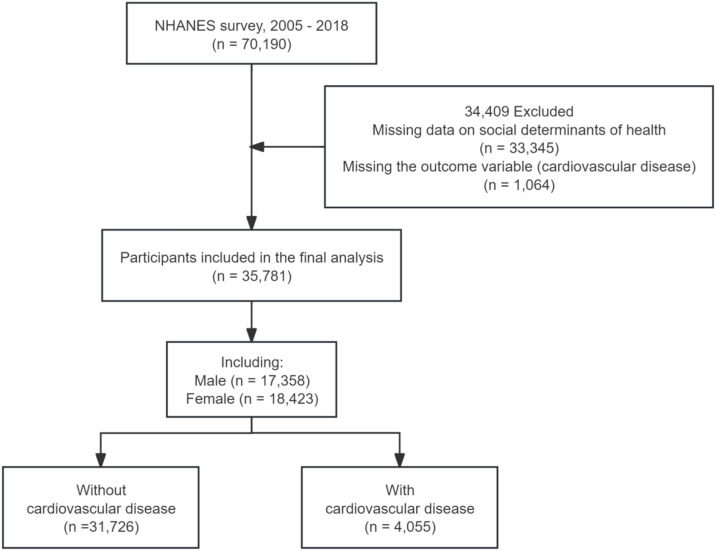
The selection process of NHANES 2005 - 2018.

Data were obtained from seven cycles of the NHANES (2005–2018), a nationally representative survey of non-institutionalized US adults. NHANES uses a multistage probability sampling design to produce nationally representative, population-weighted estimates for the non-institutionalized US population. All procedures complied with ethical standards, and written informed consent was obtained from all participants. Detailed methodological documentation, including sampling procedures and quality-control metrics, is available through the Centers for Disease Control and Prevention (CDC) NHANES website. The initial sample included 70,190 non-institutionalized participants from seven NHANES cycles conducted between 2005 and 2018. Exclusion criteria were (1) incomplete SDoH measures and (2) missing CVD status data (MCQ160B–MCQ160F). The final analytic sample comprised 35,781 participants; the selection process is shown in Fig 1.

### 2.2 Variables

#### 2.2.1 Exposure.

We operationalized SDoH using eight measures across five core domains derived from NHANES questionnaire data. We derived a composite metric by aggregating eight SDoH components to quantify cumulative exposure to adverse socioeconomic factors. This index ranges from 0 to 8, with 0 indicating no adverse SDoH exposure and ≥1 indicating at least one adverse exposure [11,12]. Each indicator was dichotomized and scored as 1 (unfavorable) or 0 (favorable): (1) employment status (unemployed vs employed, student, or retired); (2) family income-to-poverty ratio (<300% vs ≥ 300%); (3) food security (marginal, low, or very low security vs full security); (4) educational attainment (<high school vs ≥high school graduate); (5) health insurance coverage (uninsured vs insured); (6) insurance type (public insurance or no insurance vs private insurance); (7) housing tenure (renting or other arrangements vs owning a home); and (8) marital status (not married/not living with a partner vs married/living with a partner). The cumulative SDoH score (range, 0–8) was calculated as the unweighted sum of the eight indicators. We constructed the cumulative SDoH index as an unweighted additive count to represent the overall burden of co-occurring social risks across domains, consistent with cumulative-risk (or cumulative-burden) frameworks. Under this approach, the number of adverse exposures (rather than any single domain) summarizes combined social disadvantage that may jointly shape health. Unweighted additive indices are widely used in cumulative-risk research because they are parsimonious and avoid strong assumptions about the relative causal importance of heterogeneous indicators, which are often correlated and may operate through shared pathways [12,13]. We acknowledge that equal weighting is a simplifying assumption rather than a validated causal structure. Operational definitions and measurement specifications are provided in Supplementary Table 1 in S1 File.

#### 2.2.2 Outcome.

CVD status was ascertained using self-reported physician diagnoses from the NHANES Medical Conditions Questionnaire. Participants were asked, “Has a doctor or other health professional ever told you that you had (1) coronary heart disease, (2) angina pectoris, (3) myocardial infarction, or (4) a cerebrovascular event?” Participants who answered “yes” to any item were classified as having prevalent CVD.

#### 2.2.3 Covariates.

The covariates include age (20–39 years, 40–59 years, 60–79 years, or ≥80 years), sex (male or female), race (White, Black, Hispanic, and other races), and poverty-to-income ratio (PIR), which is categorized as ≥300% and <300%.

### 2.3 Statistical analysis

Following guidance from the CDC and the National Center for Health Statistics (NCHS) for complex survey analyses, we applied NHANES sampling weights. When pooling seven consecutive 2-year survey cycles, we rescaled the original 2-year Mobile Examination Center (MEC) examination weights to preserve national representativeness. Participants were stratified by CVD status (present/absent) for comparative analyses. Specifically, when combining seven 2-year cycles (2005–2006–2017–2018), we created 14-year MEC weights by dividing the 2-year MEC examination weight (WTMEC2YR) by 7, consistent with NCHS guidance for pooling cycles. The survey design was specified using masked variance units, and variances were estimated using Taylor series linearization. Continuous variables were summarized as means with standard errors (standard error (SE)). Categorical variables were summarized as weighted proportions to account for the complex survey design. Between-group comparisons were hypothesis driven: independent t-tests or analysis of variance (ANOVA) were used for continuous variables, and χ² or Fisher’s exact tests were used for categorical variables. Baseline characteristics (e.g., sex and race/ethnicity) were compared by CVD status. We assessed correlations among the eight SDoH indicators using Spearman’s rank correlation (ρ). We used survey-weighted multivariable logistic regression to quantify associations between CVD and two SDoH constructs: individual SDoH indicators and the cumulative adverse SDoH score. Models were adjusted for age, sex, and race/ethnicity. For trend tests, the cumulative SDoH score (0–8) was modeled as a continuous linear term (P for trend). For categorical analyses, each score category was compared with the reference group. Sex-by-SDoH interaction P-values (P for interaction) were obtained by adding a sex × SDoH interaction term to the corresponding model. Sensitivity analyses evaluated associations between individual SDoH indicators and CVD across the corresponding subitems. We also assessed associations between the cumulative adverse SDoH score and CVD within each of the five SDoH domains. Following prior studies, we additionally fit unweighted logistic regression models as a sensitivity analysis. In an additional model (Model 2), we further adjusted for survey cycle, body mass index (BMI), hypertension, and other covariates. Analyses were conducted in R, and statistical significance was defined as a two-tailed *P* < 0.05 (Table 1).

**Table 1:** Survey-weighted characteristic variables of the study participants stratified by cardiovascular disease, U.S. NHANES 2005–2018 (n = 35,781).

Characteristic	Total	Cardiovascular disease		*P* – value
Variable		No	Yes	
Total patients, n (%)	35781	31726 (88.67)	4055 (11.33)	< 0.0001
Cumulative number of unfavorable SDoH				< 0.0001
0	5256(22.75)	4907(23.58)	349(14.09)	
1	5940(20.78)	5325(20.85)	615(20.05)	
2	5480(15.40)	4806(15.20)	674(17.49)	
3	5022(12.68)	4430(12.66)	592(12.89)	
4	4920(10.76)	4279(10.50)	641(13.47)	
5	4524(9.28)	3965(9.09)	559(11.29)	
≥ 6	4639(8.35)	4014(8.12)	625(10.71)	
Age, years				< 0.0001
20 - 39	12284(36.96)	12121(40.03)	163(4.91)	
40 - 59	11584(37.55)	10721(38.60)	863(26.50)	
60 - 79	9516(21.23)	7388(18.45)	2128(50.23)	
≥ 80	2397(4.27)	1496(2.92)	901(18.35)	
Sex				< 0.0001
Male	17358(48.11)	15068(47.54)	2290(54.02)	
Female	18423(51.89)	16658(52.46)	1765(45.98)	
Race				< 0.0001
White	15434(67.92)	13237(67.32)	2197(74.11)	
Black	7696(11.17)	6775(11.11)	921(11.87)	
Mexiacan	5370(8.09)	4992(8.45)	378(4.27)	
Other	7281(12.82)	6722(13.12)	559(9.75)	
Employment status				< 0.0001
Employed, student, or retired	27490(80.91)	24765(81.82)	2725(71.42)	
Unemployed	8291(19.09)	6961(18.18)	1330(28.58)	
Family income-to-poverty ratio				< 0.0001
≥ 300%	12982(49.55)	11933(50.86)	1049(35.84)	
< 300%	22799(50.45)	19793(49.14)	3006(64.16)	
Food security				< 0.0001
Full security	24774(76.80)	22067(77.14)	2707(73.28)	
Marginal, low, or very low security	11007(23.20)	9659(22.86)	1348(26.72)	
Education level				< 0.0001
High school graduate or higher	27023(84.28)	24307(85.04)	2716(76.31)	
Less than high school	8758(15.72)	7419(14.96)	1339(23.69)	
Covered by health insurance				< 0.0001
Yes	28400(82.97)	24724(82.20)	3676(91.03)	
No	7381(17.03)	7002(17.80)	379(8.97)	
Type of health insurance				< 0.0001
Private	18732(62.76)	17006(63.94)	1726(50.43)	
Government or none	17049(37.24)	14720(36.06)	2329(49.57)	
Home ownership				< 0.0001
Own home	21930(67.59)	19294(67.22)	2636(71.50)	
Rent home or other arrangement	13851(32.41)	12432(32.78)	1419(28.50)	
Marital status				< 0.0001
Married or living with a partner	21264(63.40)	19096(63.84)	2168(58.82)	
Not married nor living with a partner	14517(36.60)	12630(36.16)	1887(41.18)	

Abbreviations: NHANES, National Health and Nutrition Examination Survey; SDoH, Social Determinants of Health; SE, Standard error.

## 3. Result

### 3.1 Baseline characteristics of study population

**Fig 2:**
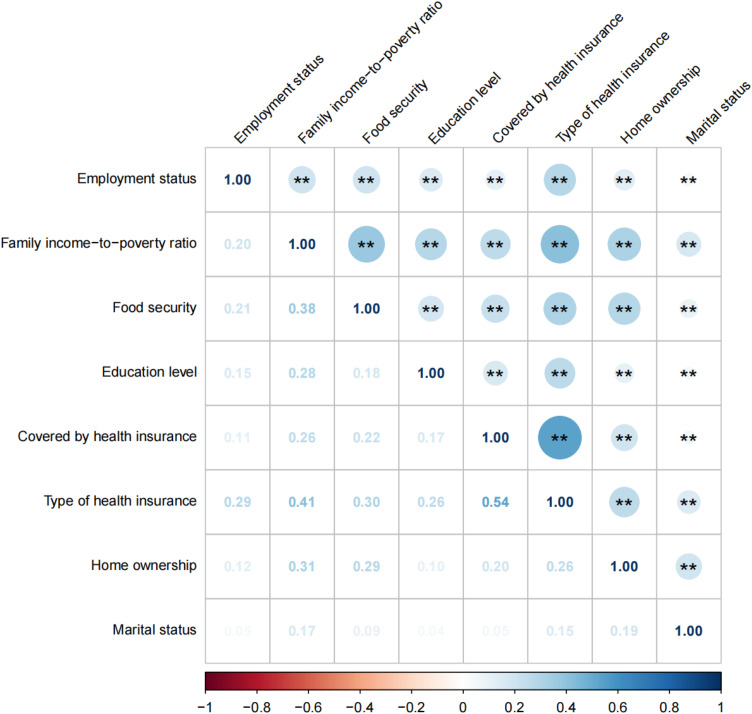
Spearman correlations between eight Social Determinants of Health variables, U.S. NHANES 2005–2018. Correlation strength was interpreted using absolute Spearman’s rho (|ρ|): < 0.10 negligible, 0.10–0.29 weak, 0.30–0.49 moderate, 0.50–0.69 strong, and ≥0.70 very strong. Positive values indicate direct correlations and negative values indicate inverse correlations.

The final analytical cohort comprised 35,781 participants (17,358 men and 18,423 women) with available cardiovascular status. Overall, 4,055 participants (11.33%) reported a clinician diagnosis of CVD, and 31,716 (88.67%) did not. Participants with CVD were older and included a higher proportion of men, White participants, and unemployed individuals (all *P* < 0.001). In addition, lower income (PIR < 300%), lower educational attainment, lack of health insurance, housing instability, and not being married or living with a partner were more common among participants with CVD (all *P* < 0.001). Descriptively, the prevalence of CVD increased across higher categories of cumulative adverse SDoH. Supplementary Tables S2–S4 in S1 File present stratified demographic distributions by sex, race, and survey cycle. Except for marital status, which showed weak correlations with other indicators, most SDoH components were positively correlated. Government insurance was strongly correlated with low income (Fig 2).

### 3.2 Association of SDoH with risk of developing CVD

Table 2 illustrates the association between SDoH and risk of developing CVD, employing a multivariable-adjusted logistic regression model to evaluate both individual SDoH domains (eight components) and cumulative adverse SDoH exposure. After controlling for demographic covariates such as age and sex, several key factors showed statistically significant associations with elevated risk of developing CVD. Upon adjusting for variables including age and sex, several factors were identified as significantly associated with an increased likelihood of developing CVD. The factors included unemployment (AOR = 2.27, 95% CI: 2.01–2.57, *P* < 0.001), a poverty income ratio (PIR) below 300% (AOR = 1.41, 95% CI: 1.25–1.58, *P* < 0.001), experiences of marginal, low, or very low food security (AOR = 1.51, 95% CI: 1.34–1.70, *P* < 0.001), and homelessness (AOR = 1.26, 95% CI: 1.12–1.41, p < 0.001). Conversely, lack of health insurance coverage was inversely associated with self-reported, clinician-diagnosed CVD (AOR = 0.70, 95% CI: 0.60–0.82, p < 0.001). The type of insurance was also found to have a significant association with CVD, with government or uninsured individuals showing a markedly increased risk (AOR = 1.38, 95% CI: 1.23–1.54, p < 0.001). Additionally, not being married or living with a partner was moderately associated with an elevated prevalence of CVD (AOR = 1.08, 95% CI: 1.08–1.09, p < 0.001), while education levels below high school did not seem to represent a significant risk factor for CVD.

**Table 2:** Comparison between different survey-weighted logistic regression models of the weighted relationship between Social Determinants of Health and cardiovascular disease, U.S. NHANES 2005–2018 (n = 35,781).

	Crude model		Model 1		Model 2	
Table 2A. Individual SDoH indicators.	COR (95% CI)	*P* – Value	AOR (95% CI)	*P* – Value	AOR (95% CI)	*P* – Value
Employment status						
Employed, student, or retired	Reference		Reference		Reference	
Unemployed	1.80(1.64,1.98)	<0.0001	3.00(2.69,3.36)	<0.0001	2.27(2.01,2.57)	<0.0001
Family income-to-poverty ratio						
≥ 300%	Reference		Reference		Reference	
< 300%	1.85(1.68,2.05)	<0.0001	2.00(1.81,2.22)	<0.0001	1.41(1.25,1.58)	<0.0001
Food security						
Full security	Reference		Reference		Reference	
Marginal, low, or very low security	1.23(1.12,1.35)	<0.0001	2.36(2.13,2.62)	<0.0001	1.51(1.34,1.70)	<0.0001
Education level						
High school graduate or higher	Reference		Reference		Reference	
Less than high school	1.76(1.59,1.96)	<0.0001	1.49(1.31,1.68)	<0.0001	1.04(0.91,1.18)	0.58
Covered by health insurance						
Yes	Reference		Reference		Reference	
No	0.45(0.39,0.52)	<0.0001	1.20(1.03,1.39)	0.02	0.70(0.60,0.82)	<0.0001
Type of health insurance						
Private	Reference		Reference		Reference	
Government or none	1.74(1.61,1.89)	<0.0001	1.89(1.73,2.07)	<0.0001	1.38(1.23,1.54)	<0.0001
Home ownership						
Own home	Reference		Reference		Reference	
Rent home or other arrangement	1.08(1.08,1.08)	<0.0001	1.76(1.60,1.95)	<0.0001	1.26(1.12,1.41)	<0.001
Marital status						
Married or living with a partner	Reference		Reference		Reference	
Not married nor living with a partner	1.24(1.13,1.35)	<0.0001	1.28(1.16,1.42)	<0.0001	1.09(1.08,1.09)	0.01
Table 2B. Cumulative number of unfavorable SDoH.						
0	Reference		Reference			
1	1.61(1.35,1.92)	<0.0001	1.45(1.21,1.74)	<0.0001		
2	1.70(1.45,2.01)	<0.0001	1.71(1.41,2.08)	<0.0001		
3	1.93(1.60,2.32)	<0.0001	1.87(1.55,2.24)	<0.0001		
4	2.08(1.74,2.49)	<0.0001	3.17(2.62,3.85)	<0.0001		
5	2.15(1.79,2.57)	<0.0001	3.94(3.24,4.79)	<0.0001		
≥ 6	2.21(1.87,2.60)	<0.0001	5.35(4.45,6.43)	<0.0001		
P for trend		<0.0001		<0.0001		

Table 2A (Individual SDoH indicators): Crude model was unadjusted. Model 1 adjusted for age, sex, and race/ethnicity. Model 2 adjusted for age, sex, race/ethnicity, and the other seven dichotomized SDoH indicators (mutual adjustment). Table 2B (Cumulative unfavorable SDoH count, 0–8): Crude model was unadjusted. Model 1 adjusted for age, sex, and race/ethnicity. Model 2 was not estimated/applicable for the cumulative SDoH count because the exposure is an aggregate count of the same indicators. Abbreviations: AOR, Adjusted odds ratio; CI, Confidence interval; COR, Crude odds ratio; NHANES, National Health and Nutrition Examination Survey; SDoH, Social Determinants of Health.

Table 2 illustrates a positive correlation between the cumulative number of adverse SDoH and the prevalence of CVD. In the fully adjusted model, the highest level of cumulative adverse SDoH (≥6 adverse SDoH sub-items) had an adjusted odds ratio (AOR) of 5.35 (95% CI: 4.45–6.43) compared to the reference level (*P* < 0.001). A notable linear trend was observed, suggesting that the prevalence of CVD rises as the accumulation of adverse SDoH increases (*P* for trend < 0.0001).

As shown in Table 3, sex-stratified survey-weighted logistic regression models were used to provide sex-specific estimates. In several comparisons, adjusted odds ratios were numerically larger in women than in men (e.g., PIR < 300%: women AOR = 1.68, 95% CI: 1.40–2.02 vs men AOR = 1.23, 95% CI: 1.07–1.41; ≥ 6 adverse SDoH: women AOR = 7.33, 95% CI: 5.43–9.90 vs men AOR = 4.39, 95% CI: 3.37–5.74). However, formal sex-by-SDoH interaction tests were not statistically significant (all *P* for interaction > 0.05); therefore, differences in point estimates should be interpreted descriptively.

**Table 3:** Association between individual and cumulative social determinants of health (SDoH) and odds of cardiovascular disease in survey-weighted logistic regression models stratified by gender, U.S. NHANES 2005–2018 (n = 35,781).

	Female (n = 18,423)				Male (n = 17,358)				P for interaction^b^
SDoH Variables	COR (95% CI)	p – Value	AOR (95% CI)	p – Value	COR (95% CI)	p – Value	AOR (95% CI)	P – Value	
Employment status									0.2378
Employed, student, or retired	Reference		Reference		Reference		Reference		
Unemployed	1.84(1.61,2.11)	**<0.0001**	2.32(1.96,2.73)	**<0.0001**	2.02(1.76,2.31)	**<0.0001**	2.20(1.87,2.60)	**<0.0001**	
Family income-to-poverty ratio									0.7536
≥ 300%	Reference		Reference		Reference		Reference		
< 300%	2.52(2.14,2.97)	**<0.0001**	1.68(1.40,2.02)	**<0.0001**	1.50(1.33,1.69)	**<0.0001**	1.23(1.07,1.41)	**0.01**	
Food security									0.5629
Full security	Reference		Reference		Reference		Reference		
Marginal, low, or very low security	1.47(1.31,1.64)	**<0.0001**	1.60(1.37,1.87)	**<0.0001**	1.05(0.91,1.21)	0.51	1.40(1.18,1.66)	**<0.001**	
Education level									0.9246
High school graduate or higher	Reference		Reference		Reference		Reference		
Less than high school	1.95(1.69,2.24)	**<0.0001**	1.06(0.94,1.16)	0.67	1.60(1.39,1.84)	<0.0001	1.13(0.94,1.37)	**0.04**	
Covered by health insurance									0.1356
Yes	Reference		Reference		Reference		Reference		
No	0.53(0.43,0.65)	**<0.0001**	0.71(0.56,0.90)	**0.01**	0.39(0.32,0.47)	<0.0001	0.73(0.59,0.89)	**0.002**	
Type of health insurance									0.6934
Private	Reference		Reference		Reference		Reference		
Government or none	1.85(1.62,2.10)	**<0.0001**	1.30(1.10,1.54)	**0.003**	1.66(1.48,1.86)	**<0.0001**	1.43(1.22,1.68)	**<0.0001**	
Home ownership									0.5823
Own home	Reference		Reference		Reference		Reference		
Rent home or other arrangement	1.10(0.97,1.25)	**<0.0001**	1.45(1.23,1.70)	**<0.0001**	1.61(1.54,1.70)	<0.0001	1.07(0.91,1.26)	0.42	
Marital status									0.1397
Married or living with a partner	Reference		Reference		Reference		Reference		
Not married nor living with a partner	1.77(1.51,2.07)	0.14	1.08(0.98,1.16)	0.48	1.04(0.92,1.24)	0.18	1.01(0.85,1.21)	0.89	
Cumulative number of unfavorable SDoH									0.2536
0	Reference		Reference		Reference		Reference		
1	2.03(1.48,2.78)	**<0.0001**	1.62(1.18,2.22)	**0.003**	1.26(1.02,1.55)	**<0.001**	1.44(1.14,1.81)	**0.002**	
2	2.84(2.08,3.88)	**<0.0001**	2.08(1.51,2.87)	**<0.0001**	1.40(1.08,1.82)	**<0.0001**	1.60(1.24,2.07)	**<0.001**	
3	2.94(2.19,3.93)	**<0.0001**	2.51(1.87,3.39)	**<0.0001**	1.50(1.17,1.92)	**0.03**	1.62(1.26,2.08)	**<0.001**	
4	3.80(2.76,5.24)	**<0.0001**	4.28(3.11,5.89)	**<0.0001**	1.55(1.25,1.92)	**<0.001**	2.73(2.11,3.54)	**<0.0001**	
5	3.86(2.86,5.21)	**<0.0001**	5.43(3.95,7.48)	**<0.0001**	1.53(1.23,1.91)	**0.01**	3.27(2.46,4.35)	**<0.0001**	
≥ 6	4.07(3.08,5.38)	**<0.0001**	7.33(5.43,9.90)	**<0.0001**	1.66(1.31,2.11)	**0.001**	4.39(3.37,5.74)	**<0.0001**	
P for trend		**<0.0001**		**<0.0001**		**0.001**		**<0.0001**	

For each of 8 dichotomized SDoH variables: Crude model was an un-adjusted model. Model 1 was adjusted for age and race. Model 2 was adjusted for age, race, and other 7 dichotomized SDoH variables; Abbreviations: AOR, Adjusted odds ratio; CI, Confidence interval; COR, Crude odds ratio; NHANES, National Health and Nutrition Examination Survey; SDoH, Social Determinants of Health.

### 3.3 Sensitivity analysis

To evaluate robustness, we conducted several sensitivity analyses (Tables S7–S9 in S1 File). First, we re-estimated models using the original multi-level categories for each SDoH indicator rather than dichotomies; the direction and statistical significance of key indicators were similar to the primary analysis (Table S7 in S1 File). Second, we constructed domain-level cumulative scores aligned with the Healthy People 2030 framework; the economic stability domain showed the strongest association with prevalent CVD, whereas several non-economic domains were attenuated after mutual adjustment (Table S8 in S1 File). Third, based on Model 2, we additionally adjusted for survey cycle and a broader set of lifestyle and clinical covariates (BMI, smoking, drinking, physical activity, hypertension, diabetes, creatinine, HDL, and total cholesterol); the associations of unemployment, low income, and higher cumulative adverse SDoH with prevalent CVD persisted (Table S9 in S1 File). Finally, unweighted logistic regression models yielded comparable patterns, suggesting that the main conclusions were not driven by survey weighting.

## 4. Discussion

Adverse SDoH indicators were associated with higher odds of prevalent CVD, underscoring the importance of economic stability. Across all models, lower income, unemployment, and food insecurity showed consistent, independent associations with prevalent CVD. Individuals experiencing multiple adverse SDoH had higher odds of prevalent CVD, and sex-stratified estimates were numerically higher among women at higher cumulative SDoH levels. Although lifestyle-related factors such as cigarette smoking and obesity are established CVD prevalence factors [14,15], their interplay with SDoH may further exacerbate cardiovascular burden [16,17].Individuals with the poverty-to-income ratio (PIR) <300% and those who were unemployed had higher odds of prevalent CVD. Although the adjusted association for unemployment (Model 2: AOR = 2.27, 95% CI = 2.01–2.57) is statistically significant, its clinical and public health significance should be interpreted in the context of the baseline prevalence. In our sample, CVD prevalence was 11.33% overall and was higher among unemployed participants (1330/8291, 16.0%) than among employed, student, or retired participants (2725/27,490, 9.9%), corresponding to an absolute difference of approximately 6.1 percentage points. Because odds ratios can overstate prevalence ratios when outcomes are not rare, the AOR should not be interpreted as a direct doubling of absolute prevalence. Using a standard conversion for common outcomes, an AOR of 2.27 corresponds to an approximate prevalence ratio of ~2.0 when the baseline prevalence is ~ 10%. Nevertheless, a roughly two-fold relative difference and a > 6 percentage-point absolute difference suggest potential clinical relevance for targeting prevention resources, consistent with prior literature linking unemployment and job insecurity to coronary heart disease and cardiovascular burden [18,19]. In addition, established lifestyle-related CVD prevalence factors are modifiable and may partially mediate the association between SDoH and CVD. A recent NHANES-linked cohort analysis reported that behavioral and clinical risk factors mediated approximately one-third of the association between unfavorable SDoH and CVD mortality, suggesting that residual SDoH-related associations may persist beyond these factors [20].

Model progression in Table 2 showed substantial attenuation or reversals in direction for some estimates, most notably for insurance coverage. For example, being uninsured (vs insured) was associated with lower odds in the crude model (OR = 0.45, 95% CI = 0.39–0.52), shifted above the null after adjustment for age, sex, and race/ethnicity (Model 1: AOR = 1.20, 95% CI = 1.03–1.39), and shifted below the null again after mutual adjustment for the other SDoH indicators (Model 2: AOR = 0.70, 95% CI = 0.60–0.82). Such changes are consistent with confounding and statistical suppression when correlated predictors are added; depending on the causal structure, mutual adjustment may also introduce overadjustment and/or reduce precision [21,22]. In addition, because CVD status in NHANES is based on self-reported clinician diagnosis, access to care may influence whether CVD is detected and subsequently reported. Prior NHANES analyses suggest that uninsured adults are more likely to have undiagnosed cardiometabolic conditions, supporting the interpretation that the crude “protective” association for uninsured status may reflect underdiagnosis or differential detection rather than truly lower disease prevalenc [18]. This pattern may also reflect differential outcome ascertainment: if underdiagnosis is more common among socioeconomically disadvantaged groups, positive associations between cumulative SDoH burden and self-reported CVD may be underestimated (biased toward the null). Conversely, access-related underdiagnosis may yield spuriously inverse associations for variables such as uninsured status, reflecting lower detection or awareness rather than true protection. Validation studies indicate that self-reported myocardial infarction and stroke have high specificity but imperfect sensitivity, and that agreement is higher among better-educated respondents, supporting potential differential misclassification by social position [23].

PIR is a key indicator of socioeconomic status, and multivariable models adjusted for age, sex, and race/ethnicity showed that PIR remained significantly associated with prevalent CVD [24,25]. Faselis et al. reported that community-dwelling older adults in the US without pre-existing cardiovascular conditions had a higher risk of incident cardiovascular events in low-income groups (hazard ratio (HR) = 1.16, 95% confidence interval (CI) = 1.03–1.31) [26]. A 17-year longitudinal cohort study further found that an income reduction of >50% was significantly associated with higher prevalence of CVD [27]. These observations are consistent with our findings and suggest that employment instability or unemployment may contribute to income loss and adverse changes in health behaviors, thereby increasing cardiovascular burden and prevalence of CVD [28].

Economically unstable, low-income populations face a higher risk of food insecurity due to budget constraints, which is associated with higher consumption of processed meats and sodium-rich foods and lower intake of fresh produce. Such dietary patterns are associated with a higher incidence of cardiometabolic diseases [29,30]. Compounding this issue, limited access to retailers offering nutrient-dense foods in low-income neighborhoods may undermine residents’ ability to maintain a balanced diet, thereby exacerbating CVD [31,32]. Even in affluent countries, low-income individuals continue to experience food shortages and unhealthy dietary patterns [33]. These unhealthy dietary patterns and associated food insecurity are linked to an increased prevalence of CVD [31,34,35]. Socioeconomic status (SES) is commonly used as an indicator of socioeconomic inequality within population [36]. Lower SES may function as a chronic stressor and is associated with inflammatory activation and atherosclerotic processes, which can contribute to cardiovascular events such as ischemic heart disease and heart failure [37–39]. Longitudinal studies suggest that socioeconomic inequality is associated with prevalence of CVD through multiple pathways [40]. Workers with lower occupational stress have more favorable cardiovascular risk profiles than those exposed to multidimensional occupational stressors, including high task demands, low autonomy, and effort–reward imbalance related to compensation, employment stability, and professional recognition (relative risk (RR) = 1.4, 95% CI = 1.1–1.8) [41]. This association remained significant after excluding earlier follow-up observations, suggesting that reverse causation (i.e., pre-existing CVD influencing stress levels) is unlikely to fully explain the findings [42].

Economic instability in low-income populations may increase prevalence of CVD directly and may also exacerbate CVD burden indirectly through factors such as educational attainment and access to health care [10,24]. Among individuals with lower income and lower educational attainment, the 10-year risk of atherosclerotic cardiovascular disease (ASCVD) events is higher [43]. Individuals with higher educational attainment may have greater health literacy and adherence to medical advice, which may facilitate preventive behaviors such as smoking cessation and healthy eating [44,45]. Economic pressure that results in housing instability may lead to prolonged psychological stress and anxiety. Chronic stress may increase stress-hormone secretion (e.g., cortisol), which may contribute to prevalence of CVD through pathways including hypertension and atherosclerosis [46]. Housing-insecure populations may be more vulnerable to cardiovascular harm due to chronic exposure to environmental stressors, including temperature extremes and airborne particulate matter [47]. Residential instability may hinder sustained health-promoting behaviors, particularly regular meals and planned physical activity. Non-homeowners may also be more likely to engage in unhealthy behaviors such as smoking and alcohol use in the context of economic instability and high stress [48]. Greater community green-space coverage may mitigate the adverse effects of air pollution on cardiovascular health [49]. In contrast, lower social cohesion and higher crime rates in socioeconomically disadvantaged neighborhoods may increase prevalence of CVD [50,51]. Furthermore, chronic environmental noise exposure has been associated with systemic inflammation, oxidative stress biomarkers, and increased susceptibility to cardiovascular disease [52,53].

Access to health care, particularly insurance coverage, may influence the observed prevalence of self-reported, clinician-diagnosed CVD. Socioeconomically disadvantaged populations frequently face structural barriers to accessing medical services, which may increase CVD prevalence [10,54]. In rural areas, identifying and addressing barriers to health care access may reduce cardiovascular events and improve outcomes [55]. After implementation of the Affordable Care Act (ACA), improvements in health coverage have been associated with fewer cardiovascular events in the US [56]. In Mexico, the Seguro Popular insurance program has been associated with higher treatment success and blood pressure control among covered patients than among uninsured patients (1.5-fold and 1.4-fold higher, respectively) [57]. Population-based studies in the US have reported that uninsured adults have higher cardiovascular risk profiles and poorer health outcomes [58]. Differences in cardiovascular outcomes by insurance type may reflect systemic variation in access to care and service quality. People with public insurance may have adequate primary care use but face barriers to specialty care and advanced treatments [57,59]. Patients with commercial insurance may have better access to newer antihypertensive medications and cardiovascular procedures, whereas those with public insurance may rely more on basic medications [60]. Furthermore, individuals with commercial or more comprehensive insurance may be more likely to receive preventive screening (e.g., blood pressure monitoring and cholesterol testing), which may contribute to better cardiovascular outcomes [61].

The inverse association for uninsured status warrants cautious interpretation. Because prevalent CVD in NHANES is based on self-reported clinician diagnosis, uninsured individuals may have fewer health care encounters and therefore lower detection and reporting, resulting in an apparently lower prevalence due to ascertainment bias. Prior studies have linked insurance coverage to preventive service use and CVD-related care utilization, and coverage expansions have been associated with improved access among adults with CVD or cardiovascular risk factors. Reverse causation and residual confounding may also contribute to this pattern.

Marital status showed limited independent associations with prevalent CVD, which may reflect the binary SDoH definition that combines heterogeneous groups. Prior evidence suggests that marital status is associated with CVD through complex, sex-dependent pathways involving social support, health behaviors, psychosocial stress, and metabolic regulation, with stronger associations reported among men [62,63]. Adjustment for correlated socioeconomic factors and access to care may further attenuate its independent association.

We observed a dose–response relationship between the cumulative count of adverse SDoH and prevalent CVD. Similar cumulative patterns have been reported in NHANES analyses using the Healthy People 2030 SDoH framework. For example, a recent cross-sectional study found that each additional adverse SDoH was associated with higher odds of premature ASCVD and reported suggestive sex differences [64].

Several pathways have been proposed to link cumulative social adversity to cardiovascular risk, including changes in health behaviors, reduced access to preventive care, and stress-related neuroendocrine or autonomic dysregulation and inflammatory activation [65]. One conceptual framework is allostatic load, defined as physiologic “wear and tear” from repeated or chronic stress exposure, which has been associated with incident CVD in prior studies [66]. However, because our analysis did not include biomarkers or longitudinal measures, these mechanistic explanations should be considered hypotheses rather than direct inferences from our data. Future studies that integrate biomarker panels and longitudinal measures are needed to test these pathways.

Sex-stratified analyses were presented to provide clinically interpretable, sex-specific estimates given the a priori relevance of sex differences in cardiovascular epidemiology. In our analyses, the association between cumulative SDoH burden and prevalent CVD appeared larger in women than in men; however, sex-by-SDoH interaction tests were not statistically significant [67]. Therefore, these sex differences should be interpreted as descriptive and exploratory. Individuals who experience long working hours and excessive work-related stress are at a notably higher risk for conditions such as stroke and atrial fibrillation [42,56,68]. Furthermore, chronic stress may contribute to autonomic imbalance, which has been linked to myocardial ischemia and arrhythmias [69,70]. Nevertheless, sex- and gender-related pathways may contribute to heterogeneity in these associations. For example, women more often assume unpaid caregiving roles and experience time poverty, which can constrain opportunities for healthy behaviors [71]. Such caregiving obligations may compromise nutritional intake through preferential selection of calorie-dense, economically constrained food options, establishing dietary patterns that could adversely affect cardiometabolic health [72]. From a pathophysiological perspective, chronic stress exposure in women appears to attenuate estrogen-mediated modulation of the HPA axis, resulting in more pronounced cortisol elevation compared to their male counterparts [73]. Concurrently, this hypercortisolemic state synergizes with IL-6 secretion to accelerate endothelial dysfunction via oxidative stress pathways and nitric oxide bioavailability reduction [74]. However, these biological pathways remain speculative in the context of our data and should be evaluated in future mechanistic studies.

Our findings indicate that cumulative social disadvantages, including unemployment, low income, and housing instability, are associated with a higher prevalence of CVD. These associations underscore that cardiovascular health is closely linked to social conditions and the distribution of resources. From a public health perspective, the strongest associations observed for economic instability point to actionable policy and clinical levers. Evidence from US health policy suggests that expanding insurance coverage is associated with improved access to care and more favorable cardiovascular outcomes among low-income adults [75]. Nutrition-support strategies that reduce food insecurity, including the Supplemental Nutrition Assistance Program (SNAP), have been linked to improved cardiometabolic risk profiles among food-insecure adults [76]. Income-support policies have also been associated with improved food security and selected cardiovascular risk factors [77]. In parallel, housing-stability interventions are increasingly recognized as relevant to cardiovascular health and may help mitigate stress-related pathways [78]. In health care settings, routine screening for social needs and referral to community resources may complement these upstream strategies. Beyond biological sex, gendered social conditions such as unpaid caregiving and time poverty may constrain opportunities for healthy behaviors and timely health care. Caregiving intensity has been linked to higher CVD risk in longitudinal studies and reviews [79,80]. Because these data reflect a pre-COVID-19 context (NHANES 2005–2018), the public-health implications should be framed as prioritizing SDoH domains and populations for contemporary evaluation rather than as evidence that specific policies will achieve comparable effects today, and the magnitude of associations should not be assumed to generalize to post-pandemic populations.

This study used nationally representative NHANES data spanning 2005–2018 (14 years). We examined five SDoH domains—economic stability, education, access to health care, community environment, and social support—and found that both individual and cumulative SDoH were associated with prevalent CVD. The cumulative SDoH score showed a dose–response association with the odds of prevalent CVD. Sensitivity analyses supported the robustness of the main findings. Although sex-stratified estimates were numerically higher among women at higher cumulative SDoH levels, interaction tests were not statistically significant; therefore, these differences should be interpreted cautiously. Taken together, these findings suggest that interventions addressing multiple social disadvantages may help inform policies aimed at improving health equity. Several limitations should be noted. First, the cross-sectional design precludes establishing temporality; adverse SDoH may precede CVD, but CVD may also contribute to income loss, unemployment, or insurance disruption. Second, SDoH indicators and CVD status were self-reported, introducing potential recall and social-desirability bias. In NHANES, CVD is based on self-reported clinician diagnosis without adjudication, allowing underreporting and misclassification; prior work also suggests that barriers to awareness and communication may further affect reporting. NHANES does not capture all upstream contextual determinants; therefore, residual confounding may persist. Insurance status and self-reported clinician diagnoses are also vulnerable to access-related ascertainment bias; thus, the inverse association for uninsured status may reflect differential detection and reverse causation rather than a lower disease burden. Third, pooled NHANES cycles from 2005–2018 represent a pre–COVID-19 context; pandemic-related disruptions and policy responses likely altered key SDoH distributions and their associations with cardiovascular health [81,82]. Finally, we lacked direct measurements of stress hormones, inflammatory markers, or vascular function. Future studies should integrate longitudinal designs with biomarker assessments to clarify mechanistic pathways.

## 5. Conclusions

This study found that adverse individual SDoH indicators and higher cumulative SDoH burden were associated with higher odds of prevalent CVD. Sex-stratified estimates suggested larger point estimates in women at higher cumulative SDoH levels; however, sex-by-SDoH interaction tests were not statistically significant. These findings emphasize the importance of integrating SDoH with the prevention and treatment of CVD, providing new perspectives and directions.

## Supporting information

S1 File Supplementary Tables S1–S9. (DOCX)

## Abbreviations

SDOH: social determinants of health
CVD: cardiovascular disease
PIR: family poverty income ratio
BMI: body mass index.
