# Supplementary material for: Individual and cumulative effects of social determinants of health on cardiovascular disease: Gender-specific insights from a cross-sectional NHANES study
Source: PLoS One. 2026 Mar 26;21(3):e0344108. doi: 10.1371/journal.pone.0344108 (PMC13020850; doi:10.1371/journal.pone.0344108)
Supplement: S1 File — (DOCX) [file pone.0344108.s001.docx]

**Supplementary Materials**

**Individual and Cumulative Effects of Social Determinants of Health on Cardiovascular Disease: Gender-Specific Insights from a Cross-sectional NHANES Study**

**Table of Contents**

**[Supplementary Table S1](#_Toc159752060)** [Specific definitions of Social Determinants of Health from Healthy People 2030 framework in U.S. NHANES 2005-2018](#_Toc159752060) **[3](#_Toc159752060)**

**[Supplementary Table S2](#_Toc159752061)** [Survey-weighted characteristic variables of the study participants according to sex, U.S. NHANES 2005-2018](#_Toc159752061) **[4](#_Toc159752061)**

**[Supplementary Table S3](#_Toc159752062)** [Survey-weighted characteristic variables of the study participants according to race, U.S. NHANES 2005-2018](#_Toc159752062) **[5](#_Toc159752062)**

**[Supplementary Table S4](#_Toc159752063)** [Survey-weighted characteristic variables of the study participants according to survey waves, U.S. NHANES 2005-2018](#_Toc159752063) **[6](#_Toc159752063)**

**[Supplementary Table S5](#_Toc159752066)** [Association between Social Determinants of Health and cardiovascular disease in survey-weighted logistic regression models stratified by race, U.S. NHANES 2005-2018](#_Toc159752066) **[7](#_Toc159752066)**

**[Supplementary Table S6](#_Toc159752067)** [Association between Social Determinants of Health and cardiovascular disease in survey-weighted logistic regression models stratified by age groups, U.S. NHANES 2005-2018](#_Toc159752067) **[8](#_Toc159752067)**

**[Supplementary Table S7](#_Toc159752068)** [Supplementary Table S7 Association between Social Determinants of Health and cardiovascular disease in survey-weighted logistic regression models using original categories, U.S. NHANES 2005-2018 ...........................................................................................9](#_Toc159752068)

**[Supplementary Table S8](#_Toc159752069)** [Association between five domains about Social Determinants of Health and cardiovascular disease in survey-weighted logistic regression models, U.S. NHANES 2005-2018](#_Toc159752069) **[1](#_Toc159752069)0**

**[Supplementary Table S9](#_Toc159752070)** [Further adjustments in sensitivity analyses for Social Determinants of Health associated with cardiovascular disease by survey-weighted logistic regression models, U.S. NHANES 2005-2018](#_Toc159752070) **[1](#_Toc159752070)1**

**Supplementary Table S1 Specific definitions of Social Determinants of Health from Healthy People 2030 framework in U.S. NHANES 2005-2018**

| **Domain** | **Subdomain** | **Dichotomized categories** | **Original categories** | **Definitions** |
| --- | --- | --- | --- | --- |
| **Economic Stability** | Employment status | 0: Employed, student, or retired 1: Unemployed | Employed, student, or retired Unemployed | Participants were asked questions about their work experience, including the type of work done last week or the main reason did not work last week, and other job-related questions. With the exception on students and retirees, all participants who are not employed were identified as unemployed. |
|  | Family income-to-poverty ratio | 0: ≥ 300% 1: < 300% | > 500%  300-500% 100-300% < 100% | Participants were asked their total family income and total number of people in the family to evaluate the ratio of family income to poverty guidelines, which was calculated by dividing family income by a family size-specific poverty threshold. |
|  | Food security | 0: Full security 1: Marginal, low, or very low security | Full security (0 affirmative) Marginal security (1-2 affirmative) Low security (3-5 affirmative) Very low security (6-10 affirmative) | In the household interview, participants responded to 10 items of the US FSSM questions (Bickel G, et al, 2000), which refer to all household members, not just NHANES participants. These 10 items were: In the last 12 months, 1) they were worried about the food would run out before they got money to buy more; 2) the food that they bought just didn’t last, and they didn’t have money to get more; 3) they couldn’t afford to eat balanced meals; 4) they had ever cut the size of meals or skip meals because there wasn’t enough money for food; 5) how often they had ever cut the size of meals or skip meals because there wasn’t enough money for food; 6) they had ever eat less than they felt they should because there wasn’t enough money to buy food; 7) they were ever hungry but didn’t eat because they couldn’t afford enough food; 8) they lost weight because they didn’t have enough money for food; 9) they had ever not eat for a whole day because there wasn’t enough money for food; 10) how often they had ever not eat for a whole day because there wasn’t enough money for food. The participants’ responses total 10 items were categorized into four levels, which were created based on the cumulative number of affirmative responses to these items: Full food security (no affirmative response in any of these items), Marginal food security (1-2 affirmative responses), Low food security (3-5 affirmative responses), Very low food security (6-10 affirmative responses). |
| **Education Access and Quality** | Education level | 0: High school graduate or higher 1: Less than high school | College Some college or AA degree High school Less than high school | Participants were asked about the highest grade or level of school they completed or the highest degree they received. Participants’ responses were categorized into five categories: Less than 9th grade, 9-11th grade (Includes 12th grade with no diploma), High school graduate/GED or equivalent, Some college or AA degree, College graduate or above. |
| **Health Care Access and Quality** | Health insurance coverage | 0: Yes 1: No | Yes No | Participants were asked whether they are covered by health insurance or some other kind of health care plan (Include health insurance obtained through employment or purchased directly as well as government programs like Medicare and Medicaid that provide medical care or help pay medical bills.) |
|  | Type of health insurance | 0: Private 1: Government or none | Private insurance Government insurance No insurance | Participants were further asked about the type of insurance, including private insurance and some kinds of government insurance (such as Medicare, Medi-Gap, Medicaid, CHIP/SCHIP, military health care, Indian Health Service, state-sponsored health plan, single service plan, or other government insurance). |
| **Neighborhood and Built Environment** | Home ownership | 0: Own home 1: Rent home or other arrangement | Own home Rent home Other arrangement | Participants were asked whether the home they are living in is owned, being bought, rented, or occupied by some other arrangement by themself or someone else in their family. |
| **Social and Community Context** | Marital status | 0: Married or living with a partner 1: Not married nor living with a partner | Married or living with a partner Never married Widowed Divorced Separated | Participants were asked whether they were married, widowed, divorced, separated, never married, or living with partner. Those participants who were married or living with partner were grouped into one category. |

| Footnotes: SDoH are the conditions in the environments where people are born, live, learn, work, play, worship, and age that affect a wide range of health, functioning, and quality-of-life outcomes and risks. According to Healthy People 2030 framework, SDoH can be grouped into 5 domains: Economic Stability, Education Access and Quality, Health Care Access and Quality, Neighborhood and Built Environment, and Social and Community Context.  Abbreviations: AA, Associate's Degree; CHIP, Children’s Health Insurance Program; GED, General equivalent diploma; NHANES, National Health and Nutrition Examination Survey; US FSSM, U.S. Food Security Survey Module; SCHIP, State Children's Health Insurance Program; SDoH, Social determinants of health. References: [1] Bickel G, Nord M, Price C, Hamilton W, Cook J. Guide to Measuring Household Food Security Revised 2000. U.S. Department of Agriculture, Food and Nutrition Service, Alexandria VA. March, 2000. Available from: http://www.fns.usda.gov/sites/default/files/FSGuide_0.pdf [2] US Department of Health and Human Services. Healthy People 2030: social determinants of health. https://health.gov/healthypeople/objectives-and-data/social-determinants-health [3] Gómez CA, Kleinman DV, Pronk N, et al. Addressing Health Equity and Social Determinants of Health Through Healthy People 2030. J Public Health Manag Pract. 2021;27 (Suppl 6):S249-S257. doi:10.1097/PHH.0000000000001297 |
| --- |

**Supplementary Table S2 Survey-weighted characteristic variables of the study participants according to sex, U.S. NHANES 2005-2018**

| Characteristic | Total | Sex | | | *P* - value^b^ |
| --- | --- | --- | --- | --- | --- |
| Variable |  | Female | | Male |  |
| Total patients, n (%) | 35781 | | 18423(51.89) | 17358(48.11) |  |
| Age, years |  | |  |  |  |
| 20 - 39 | 12284(36.96) | | 6440(36.06) | 5844(37.92) | **< 0.0001** |
| 40 - 59 | 11584(37.55) | | 6006(37.03) | 5578(38.10) |  |
| 60 - 79 | 9516(21.23) | | 4719(21.86) | 4797(20.55) |  |
| ≥ 80 | 2397( 4.27) | | 1258(5.05) | 1139(3.42) |  |
| Race |  | |  |  | **< 0.0001** |
| White | 15434(67.92) | | 7808(67.72) | 7626(68.13) |  |
| Black | 7696(11.17) | | 3999(11.95) | 3697(10.33) |  |
| Mexiacan | 5370( 8.09) | | 2754(7.42) | 2616(8.81) |  |
| Other | 7281(12.82) | | 3862(12.91) | 3419(12.73) |  |
| Employment status |  | |  |  | **< 0.0001** |
| Employed, student, or retired | 27490(80.91) | | 13139(75.67) | 14351(86.56) |  |
| Unemployed | 8291(19.09) | | 5284(24.33) | 3007(13.44) |  |
| Family income-to-poverty ratio |  | |  |  | **< 0.0001** |
| ≥ 300% | 12982(49.55) | | 6384(47.42) | 6598(51.84) |  |
| < 300% | 22799(50.45) | | 12039(52.58) | 10760(48.16) |  |
| Food security |  | |  |  | **< 0.0001** |
| Full security | 24774(76.80) | | 12542(75.89) | 12232(77.78) |  |
| Marginal, low, or very low security | 11007(23.20) | | 5881(24.11) | 5126(22.22) |  |
| Education level |  | |  |  | **0.001** |
| High school graduate or higher | 27023(84.28) | | 14132(85.06) | 12891(83.43) |  |
| Less than high school | 8758(15.72) | | 4291(14.94) | 4467(16.57) |  |
| Covered by health insurance |  | |  |  | **< 0.0001** |
| Yes | 28400(82.97) | | 15012(85.24) | 13388(80.52) |  |
| No | 7381(17.03) | | 3411(14.76) | 3970(19.48) |  |
| Type of health insurance |  | |  |  | 0.31 |
| Private | 18732(62.76) | | 9679(63.02) | 9053(62.47) |  |
| Government or none | 17049(37.24) | | 8744(36.98) | 8305(37.53) |  |
| Home ownership |  | |  |  | 0.87 |
| Own home | 21930(67.59) | | 11140(67.63) | 10790(67.56) |  |
| Rent home or other arrangement | 13851(32.41) | | 7283(32.37) | 6568(32.44) |  |
| Marital status |  | |  |  | **< 0.0001** |
| Married or living with a partner | 21264(63.40) | | 10022(60.14) | 11242(66.91) |  |
| Not married nor living with a partner | 14517(36.60) | | 8401(39.86) | 6116(33.09) |  |
| Cardiovascular disease |  | |  |  | **< 0.0001** |
| No | 31726(91.25) | | 16658(92.24) | 15068(90.17) |  |
| Yes | 4055(8.75) | | 1765(7.76) | 2290(9.83) |  |
| Cumulative number of unfavorable SDoH^c^ |  | |  |  | **< 0.0001** |
| 0 | 5256(22.75) | | 2373(20.31) | 2883(25.37) |  |
| 1 | 5940(20.78) | | 3014(21.17) | 2926(20.36) |  |
| 2 | 5480(15.40) | | 2825(15.96) | 2655(14.79) |  |
| 3 | 5022(12.68) | | 2661(13.26) | 2361(12.06) |  |
| 4 | 4920(10.76) | | 2559(11.02) | 2361(10.48) |  |
| 5 | 4524(9.28) | | 2400(9.52) | 2124(9.02) |  |
| ≥ 6 | 4639(8.35) | | 2591(8.74) | 2048(7.93) |  |

Footnotes: Continuous variables are presented as weighted mean ± SE, and categorical variables are presented as counting (n) and survey-weighted percentage (%).

Abbreviations: NHANES, National Health and Nutrition Examination Survey; SDoH, Social Determinants of Health; SE, Standard error.

^a^Age-adjusted prevalence rates are present as [% (SE)]. There were two steps to calculate them: First, the standard age proportions for age groups, based on the 2000 U.S Census Standard Population data, were calculated by dividing the age-specific Census population (P) by the total Census population number (T), and the standardizing proportions (P/T) should sum to 1. Second, the age-specific prevalence from the study population is multiplied by the proportion of people in that age group in the standard population, and results summed up to get the age-adjusted estimates. More detail can be got from: https://wwwn.cdc.gov/nchs/nhanes/tutorials/samplecode.aspx.

^b^The P-values were assessed by t-test (continuous variables) or by chi-square test (categorical variables) to represent the differences of participants with and without urge urinary incontinence. P-values presented with bold valued were statistically significant with P-value < 0.05.

^c^Cumulative number of unfavorable SDoH was calculated by accumulating the 8 dichotomized SDoH with a value of 0 for favorable level and a value of 1 for unfavorable level.

**Supplementary Table S3 Survey-weighted characteristic variables of the study participants according to race, U.S. NHANES 2005-2018**

| Characteristic | Total | Race | | | | *P* - value^b^ |
| --- | --- | --- | --- | --- | --- | --- |
| Variable |  | White | Black | Mexican | Other |  |
| Total patients, n (%) | 35781 | 15434(67.92) | 7696(11.17) | 5370(8.09) | 7281(12.82) |  |
| Age, years |  |  |  |  |  |  |
| 20 - 39 | 12284(36.96) | 4792(32.55) | 2589(42.01) | 2177(53.46) | 2726(45.47) | **< 0.0001** |
| 40 - 59 | 11584(37.55) | 4665(37.98) | 2593(38.17) | 1773(34.13) | 2553(36.83) |  |
| 60 - 79 | 9516(21.23) | 4217(24.14) | 2225(17.30) | 1301(11.21) | 1773(15.60) |  |
| ≥ 80 | 2397( 4.27) | 1760(5.33) | 289(2.52) | 119(1.19) | 229(2.10) |  |
| Sex |  |  |  |  |  | **< 0.0001** |
| Male | 17358(48.11) | 7626(48.26) | 3697(44.48) | 2616(52.39) | 3419(47.77) |  |
| Female | 18423(51.89) | 7808(51.74) | 3999(55.52) | 2754(47.61) | 3862(52.23) |  |
| Employment status |  |  |  |  |  | **< 0.0001** |
| Employed, student, or retired | 27490(80.91) | 12299(83.12) | 5757(74.92) | 3891(75.64) | 5543(77.71) |  |
| Unemployed | 8291(19.09) | 3135(16.88) | 1939(25.08) | 1479(24.36) | 1738(22.29) |  |
| Family income-to-poverty ratio |  |  |  |  |  | **< 0.0001** |
| ≥ 300% | 12982(49.55) | 6735(57.32) | 2465(32.43) | 1060(21.78) | 2722(40.79) |  |
| < 300% | 22799(50.45) | 8699(42.68) | 5231(67.57) | 4310(78.22) | 4559(59.21) |  |
| Food security |  |  |  |  |  | **< 0.0001** |
| Full security | 24774(76.80) | 12073(83.57) | 4858(61.89) | 2908(53.70) | 4935(68.52) |  |
| Marginal, low, or very low security | 11007(23.20) | 3361(16.43) | 2838(38.11) | 2462(46.30) | 2346(31.48) |  |
| Education level |  |  |  |  |  | **< 0.0001** |
| High school graduate or higher | 27023(84.28) | 12996(89.45) | 5928(79.55) | 2595(54.83) | 5504(79.59) |  |
| Less than high school | 8758(15.72) | 2438(10.55) | 1768(20.45) | 2775(45.17) | 1777(20.41) |  |
| Covered by health insurance |  |  |  |  |  | **< 0.0001** |
| Yes | 28400(82.97) | 13300(88.09) | 6189(77.77) | 3163(55.28) | 5748(77.81) |  |
| No | 7381(17.03) | 2134(11.91) | 1507(22.23) | 2207(44.72) | 1533(22.19) |  |
| Type of health insurance |  |  |  |  |  | **< 0.0001** |
| Private | 18732(62.76) | 9578(70.21) | 3600(47.23) | 1876(36.67) | 3678(53.25) |  |
| Government or none | 17049(37.24) | 5856(29.79) | 4096(52.77) | 3494(63.33) | 3603(46.75) |  |
| Home ownership |  |  |  |  |  | **< 0.0001** |
| Own home | 21930(67.59) | 11065(74.99) | 3870(47.52) | 3050(53.29) | 3945(54.95) |  |
| Rent home or other arrangement | 13851(32.41) | 4369(25.01) | 3826(52.48) | 2320(46.71) | 3336(45.05) |  |
| Marital status |  |  |  |  |  | **< 0.0001** |
| Married or living with a partner | 21264(63.40) | 9536(65.86) | 3438(44.15) | 3607(67.46) | 4683(64.60) |  |
| Not married nor living with a partner | 14517(36.60) | 5898(34.14) | 4258(55.85) | 1763(32.54) | 2598(35.40) |  |
| Chronic kidney disease |  |  |  |  |  | **< 0.0001** |
| No | 31726(91.25) | 13237(90.45) | 6775(90.70) | 4992(95.38) | 6722(93.34) |  |
| Yes | 4055( 8.75) | 2197(9.55) | 921(9.30) | 378(4.62) | 559(6.66) |  |
| Cumulative number of unfavorable SDoH^c^ |  |  |  |  |  | **< 0.0001** |
| 0 | 5256(22.75) | 3162(28.22) | 754( 9.50) | 345( 7.01) | 995(15.22) |  |
| 1 | 5940(20.78) | 3265(24.13) | 1033(13.41) | 496( 9.84) | 1146(16.38) |  |
| 2 | 5480(15.40) | 2631(16.02) | 1117(14.08) | 584(10.79) | 1148(16.18) |  |
| 3 | 5022(12.68) | 2149(12.04) | 1133(14.65) | 691(12.78) | 1049(14.31) |  |
| 4 | 4920(10.76) | 1696( 8.60) | 1226(15.91) | 943(17.31) | 1055(13.59) |  |
| 5 | 4524( 9.28) | 1391( 6.51) | 1136(15.07) | 1023(18.74) | 974(12.95) |  |
| ≥ 6 | 4639( 8.35) | 1140( 4.49) | 1297(17.38) | 1288(23.54) | 914(11.37) |  |

Footnotes: Continuous variables are presented as weighted mean ± SE, and categorical variables are presented as counting (n) and survey-weighted percentage (%).

Abbreviations: NHANES, National Health and Nutrition Examination Survey; SDoH, Social Determinants of Health; SE, Standard error.

^a^Age-adjusted prevalence rates are present as [% (SE)]. There were two steps to calculate them: First, the standard age proportions for age groups, based on the 2000 U.S Census Standard Population data, were calculated by dividing the age-specific Census population (P) by the total Census population number (T), and the standardizing proportions (P/T) should sum to 1. Second, the age-specific prevalence from the study population is multiplied by the proportion of people in that age group in the standard population, and results summed up to get the age-adjusted estimates. More detail can be got from: https://wwwn.cdc.gov/nchs/nhanes/tutorials/samplecode.aspx.

^b^The P-values were assessed by t-test (continuous variables) or by chi-square test (categorical variables) to represent the differences of participants with and without urge urinary incontinence. P-values presented with bold valued were statistically significant with P-value < 0.05.

^c^Cumulative number of unfavorable SDoH was calculated by accumulating the 8 dichotomized SDoH with a value of 0 for favorable level and a value of 1 for unfavorable level.

**Supplementary Table S4 Survey-weighted characteristic variables of the study participants according to survey waves, U.S. NHANES 2005-2018 (n = 31,332)**

| SDoH | Total | Survey waves | | | | | | | *P* - value^b^ |
| --- | --- | --- | --- | --- | --- | --- | --- | --- | --- |
| Variables |  | 2005 - 2006 | 2007 - 2008 | 2009 - 2010 | 2011 - 2012 | 2013 - 2014 | 2015 - 2016 | 2017 - 2018 |  |
| Total patients, n (%) | 35781 | 4709(13.93) | 5353(13.68) | 5570(13.87) | 5052(14.47) | 5290(14.75) | 5049(14.79) | 4758(14.52) |  |
| Age, years |  |  |  |  |  |  |  |  |  |
| 20 - 39 | 12284(36.96) | 1832(38.29) | 1740(37.68) | 1883(37.15) | 1814(36.65) | 1807(36.19) | 1758(36.40) | 1450(36.45) | 0.09 |
| 40 - 59 | 11584(37.55) | 1421(39.30) | 1698(39.26) | 1850(38.37) | 1653(38.01) | 1799(36.88) | 1677(36.81) | 1486(34.41) |  |
| 60 - 79 | 9516(21.23) | 1104(18.40) | 1533(18.76) | 1460(20.10) | 1278(21.03) | 1364(22.56) | 1316(22.70) | 1461(24.72) |  |
| ≥ 80 | 2397( 4.27) | 352(4.01) | 382(4.30) | 377(4.38) | 307(4.31) | 320(4.36) | 298(4.09) | 361(4.42) |  |
| Sex |  |  |  |  |  |  |  |  | 1.00 |
| Male | 17358(48.11) | 2258(48.09) | 2630(48.04) | 2714(48.34) | 2480(48.05) | 2525(48.07) | 2438(47.94) | 2313(48.24) |  |
| Female | 18423(51.89) | 2451(51.91) | 2723(51.96) | 2856(51.66) | 2572(51.95) | 2765(51.93) | 2611(52.06) | 2445(51.76) |  |
| Race |  |  |  |  |  |  |  |  | 0.72 |
| White | 15434(67.92) | 2374(72.13) | 2551(70.24) | 2786(69.67) | 1927(67.60) | 2324(66.73) | 1718(65.32) | 1754(64.16) |  |
| Black | 7696(11.17) | 1075(11.50) | 1117(11.12) | 1002(11.08) | 1299(11.13) | 1078(11.33) | 1047(11.00) | 1078(11.05) |  |
| Mexican | 5370( 8.09) | 917(7.63) | 907(8.14) | 960(8.06) | 479(7.31) | 663(8.68) | 853(8.43) | 591(8.33) |  |
| Other | 7281(12.82) | 343( 8.73) | 778(10.50) | 822(11.18) | 1347(13.96) | 1225(13.26) | 1431(15.24) | 1335(16.47) |  |
| Employment status |  |  |  |  |  |  |  |  | 0.08 |
| Employed, student, or retired | 27490(80.91) | 3701(83.08) | 4163(81.21) | 4165(79.32) | 3791(78.91) | 4074(79.84) | 3883(81.83) | 3713(82.17) |  |
| Unemployed | 8291(19.09) | 1008(16.92) | 1190(18.79) | 1405(20.68) | 1261(21.09) | 1216(20.16) | 1166(18.17) | 1045(17.83) |  |
| Family income-to-poverty ratio |  |  |  |  |  |  |  |  | 0.53 |
| ≥ 300% | 12982(49.55) | 1965(53.39) | 1868(48.87) | 1961(49.61) | 1768(47.06) | 1994(48.10) | 1680(48.47) | 1746(51.48) |  |
| < 300% | 22799(50.45) | 2744(46.61) | 3485(51.13) | 3609(50.39) | 3284(52.94) | 3296(51.90) | 3369(51.53) | 3012(48.52) |  |
| Food security |  |  |  |  |  |  |  |  | **< 0.0001** |
| Full security | 24774(76.80) | 3662(84.23) | 3975(81.20) | 3827(77.60) | 3422(75.29) | 3731(76.03) | 3110(71.87) | 3047(72.07) |  |
| Marginal, low, or very low security | 11007(23.20) | 1047(15.77) | 1378(18.80) | 1743(22.40) | 1630(24.71) | 1559(23.97) | 1939(28.13) | 1711(27.93) |  |
| Education level |  |  |  |  |  |  |  |  | **< 0.0001** |
| High school graduate or higher | 27023(84.28) | 3417(82.68) | 3701(79.75) | 4045(81.55) | 3897(84.20) | 4200(85.35) | 3894(86.31) | 3869(89.59) |  |
| Less than high school | 8758(15.72) | 1292(17.32) | 1652(20.25) | 1525(18.45) | 1155(15.80) | 1090(14.65) | 1155(13.69) | 889(10.41) |  |
| Covered by health insurance |  |  |  |  |  |  |  |  | **< 0.001** |
| Yes | 28400(82.97) | 3712(82.01) | 4115(81.40) | 4234(80.18) | 3859(80.11) | 4194(82.27) | 4196(87.14) | 4090(87.33) |  |
| No | 7381(17.03) | 997(17.99) | 1238(18.60) | 1336(19.82) | 1193(19.89) | 1096(17.73) | 853(12.86) | 668(12.67) |  |
| Type of health insurance |  |  |  |  |  |  |  |  | 0.24 |
| Private | 18732(62.76) | 2651(65.54) | 2868(65.52) | 2912(63.42) | 2538(61.02) | 2786(61.71) | 2574(62.69) | 2403(59.72) |  |
| Government or none | 17049(37.24) | 2058(34.46) | 2485(34.48) | 2658(36.58) | 2514(38.98) | 2504(38.29) | 2475(37.31) | 2355(40.28) |  |
| Home ownership |  |  |  |  |  |  |  |  | 0.05 |
| Own home | 21930(67.59) | 3147(72.67) | 3494(70.38) | 3444(68.71) | 2841(63.11) | 3154(65.70) | 2980(66.83) | 2870(66.20) |  |
| Rent home or other arrangement | 13851(32.41) | 1562(27.33) | 1859(29.62) | 2126(31.29) | 2211(36.89) | 2136(34.30) | 2069(33.17) | 1888(33.80) |  |
| Marital status |  |  |  |  |  |  |  |  | 0.45 |
| Married or living with a partner | 21264(63.40) | 2955(65.89) | 3188(63.63) | 3293(63.25) | 2839(61.40) | 3104(62.30) | 3068(64.65) | 2817(62.77) |  |
| Not married nor living with a partner | 14517(36.60) | 1754(34.11) | 2165(36.37) | 2277(36.75) | 2213(38.60) | 2186(37.70) | 1981(35.35) | 1941(37.23) |  |
| Chronic kidney disease |  |  |  |  |  |  |  |  | 0.93 |
| No | 31726(91.25) | 4190(91.50) | 4691(91.28) | 4937(91.41) | 4515(91.23) | 4743(91.16) | 4496(91.56) | 4154(90.60) |  |
| Yes | 4055( 8.75) | 519(8.50) | 662(8.72) | 633(8.59) | 537(8.77) | 547(8.84) | 553(8.44) | 604(9.40) |  |
| Cumulative number of unfavorable SDoH^c^ |  |  |  |  |  |  |  |  | 0.12 |
| 0 | 5256(22.75) | 845(25.43) | 837(24.32) | 825(22.93) | 668(20.41) | 813(22.20) | 638(22.81) | 630(21.35) |  |
| 1 | 5940(20.78) | 874(22.58) | 872(20.86) | 885(20.71) | 787(20.37) | 904(19.82) | 782(19.74) | 836(21.49) |  |
| 2 | 5480(15.40) | 742(15.88) | 826(14.60) | 837(15.34) | 709(14.64) | 805(15.59) | 801(15.78) | 760(15.91) |  |
| 3 | 5022(12.68) | 629(11.77) | 740(12.66) | 732(11.66) | 704(12.60) | 710(12.24) | 794(14.21) | 713(13.54) |  |
| 4 | 4920(10.76) | 569( 9.49) | 676( 9.61) | 702(10.02) | 747(11.12) | 741(11.67) | 769(11.29) | 716(11.94) |  |
| 5 | 4524( 9.28) | 537( 8.21) | 644( 8.78) | 743( 9.74) | 711(11.03) | 648( 9.47) | 637( 8.43) | 604( 9.27) |  |
| ≥ 6 | 4639( 8.35) | 513(6.65) | 758(9.16) | 846(9.61) | 726(9.82) | 669(9.02) | 628(7.75) | 499(6.49) |  |

Footnotes: Continuous variables are presented as weighted mean ± SE, and categorical variables are presented as counting (n) and survey-weighted percentage (%).

Abbreviations: NHANES, National Health and Nutrition Examination Survey; SDoH, Social Determinants of Health; SE, Standard error.

^a^Age-adjusted prevalence rates are present as [% (SE)]. There were two steps to calculate them: First, the standard age proportions for age groups, based on the 2000 U.S Census Standard Population data, were calculated by dividing the age-specific Census population (P) by the total Census population number (T), and the standardizing proportions (P/T) should sum to 1. Second, the age-specific prevalence from the study population is multiplied by the proportion of people in that age group in the standard population, and results summed up to get the age-adjusted estimates. More detail can be got from: https://wwwn.cdc.gov/nchs/nhanes/tutorials/samplecode.aspx.

^b^The P-values were assessed by t-test (continuous variables) or by chi-square test (categorical variables) to represent the differences of participants with and without urge urinary incontinence. P-values presented with bold valued were statistically significant with P-value < 0.05.

^c^Cumulative number of unfavorable SDoH was calculated by accumulating the 8 dichotomized SDoH with a value of 0 for favorable level and a value of 1 for unfavorable level.

**Supplementary Table S5 Association between Social Determinants of Health and cardiovascular disease in survey-weighted logistic regression models stratified by race, U.S. NHANES 2005-2018**

| Characteristic | White | | Black | | Mexican | | Other | |
| --- | --- | --- | --- | --- | --- | --- | --- | --- |
| Variable | COR (95% CI) | AOR (95% CI) | COR (95% CI) | AOR (95% CI) | COR (95% CI) | AOR (95% CI) | COR (95% CI) | AOR (95% CI) |
| Employment status |  |  |  |  |  |  |  |  |
| Employed, student, or retired | Reference | Reference | Reference | Reference | Reference | Reference | Reference | Reference |
| Unemployed | **1.60(1.41,1.82)^**^** | **2.06(1.72,2.45)^**^** | **2.77(2.34,3.27)^**^** | **2.60(2.15,3.15)^**^** | **2.76(2.14,3.56)^**^** | **2.58(1.97,3.37)^**^** | **2.35(1.79,3.08)^**^** | **2.67(1.88,3.77)^**^** |
| Family income-to-poverty ratio |  |  |  |  |  |  |  |  |
| ≥ 300% | Reference | Reference | Reference | Reference | Reference | Reference | Reference | Reference |
| < 300% | **2.19(1.94,2.46)^**^** | **1.46(1.26,1.69)^**^** | **1.70(1.36,2.13)^**^** | **1.18(0.91,1.53)^*^** | **1.58(1.06,2.36)^**^** | 1.23(0.75,2.00) | **1.63(1.21,2.20)^**^** | 1.17(0.79,1.72) |
| Food security |  |  |  |  |  |  |  |  |
| Full security | Reference | Reference | Reference | Reference | Reference | Reference | Reference | Reference |
| Marginal, low, or very low security | **1.42(1.24,1.62)^**^** | **1.67(1.42,1.97)^**^** | **1.31(1.13,1.53)^**^** | **1.25(1.03,1.51)^*^** | 1.17(0.92,1.48) | 1.15(0.84,1.57) | **1.34(1.07,1.67)^*^** | **1.37(1.03,1.82)^*^** |
| Education level |  |  |  |  |  |  |  |  |
| High school graduate or higher | Reference | Reference | Reference | Reference | Reference | Reference | Reference | Reference |
| Less than high school | **2.27(1.95,2.64)^**^** | **1.18(0.99,1.40)^**^** | **2.07(1.78,2.41)^**^** | 1.07(0.90,1.27) | **1.55(1.19,2.01)^**^** | 1.12(0.91,1.39) | **1.81(1.39,2.35)^**^** | 0.86(0.64,1.15) |
| Covered by health insurance |  |  |  |  |  |  |  |  |
| Yes | Reference | Reference | Reference | Reference | Reference | Reference | Reference | Reference |
| No | **0.51(0.42,0.63)^**^** | **0.75(0.60,0.93)^*^** | **0.42(0.34,0.52)^**^** | **0.49(0.37,0.63)^**^** | **0.46(0.34,0.60)^**^** | **0.66(0.48,0.90)^*^** | **0.56(0.39,0.81)^**^** | 0.70(0.46,1.07) |
| Type of health insurance |  |  |  |  |  |  |  |  |
| Private | Reference | Reference | Reference | Reference | Reference | Reference | Reference | Reference |
| Government or none | **1.92(1.73,2.13)^**^** | **1.33(1.16,1.53)^**^** | **1.96(1.69,2.27)^**^** | **1.63(1.34,1.98)^**^** | **1.79(1.33,2.41)^**^** | **1.71(1.18,2.48)^**^** | **2.27(1.63,3.16)^**^** | **1.56(1.01,2.41)^*^** |
| Home ownership |  |  |  |  |  |  |  |  |
| Own home | Reference | Reference | Reference | Reference | Reference | Reference | Reference | Reference |
| Rent home or other arrangement | **1.26(1.15,1.38)^**^** | **1.33(1.13,1.58)^**^** | **1.04(0.88,1.24)^*^** | **1.19(0.98,1.44)^*^** | **1.30(1.07,1.58)^*^** | 1.21(0.91,1.60) | **1.50(1.21,1.85)^*^** | **1.14(0.91,1.42)^*^** |
| Marital status |  |  |  |  |  |  |  |  |
| Married or living with a partner | Reference | Reference | Reference | Reference | Reference | Reference | Reference | Reference |
| Not married nor living with a partner | **1.27(1.13,1.42)^**^** | 1.21(1.06,1.39) | **1.21(1.01,1.43)^*^** | 1.12(0.96,1.29) | **1.31(1.03,1.66)^*^** | 1.08(0.80,1.45) | 1.01(0.80,1.27) | **1.13(0.92,1.38)^*^** |
|  |  |  |  |  |  |  |  |  |
| Cumulative number of unfavorable SDoH |  |  |  |  |  |  |  |  |
| 0 | Reference | Reference | Reference | Reference | Reference | Reference | Reference | Reference |
| 1 | **1.64(1.36,1.97)^**^** | **1.41(1.17,1.71)^**^** | **1.40(1.00,1.95)^*^** | **1.47(1.05,2.06)^*^** | 1.89(0.81,4.42) | 1.99(0.78,5.05) | 1.85(0.98,3.49) | 1.75(0.92,3.33) |
| 2 | **2.13(1.74,2.61)^**^** | **1.70(1.36,2.12)^**^** | **1.55(1.05,2.30)^*^** | **1.60(1.07,2.38)^*^** | 1.80(0.93,3.51) | 1.92(0.94,3.93) | 1.85(0.97,3.52) | 1.68(0.88,3.20) |
| 3 | **1.91(1.57,2.31)^**^** | **1.87(1.51,2.32)^**^** | **1.83(1.31,2.56)^**^** | **2.04(1.46,2.86)^**^** | 1.93(0.86,4.35) | 1.90(0.82,4.43) | 1.50(0.79,2.85) | 1.41(0.74,2.66) |
| 4 | **2.66(2.14,3.31)^**^** | **3.60(2.81,4.62)^**^** | **1.94(1.32,2.87)^**^** | **2.34(1.57,3.49)^**^** | **3.41(1.54,7.58)^**^** | **3.87(1.69,8.82)^**^** | **1.80(1.02,3.19)^*^** | **1.87(1.07,3.29)^*^** |
| 5 | **2.45(1.95,3.06)^**^** | **4.30(3.33,5.54)^**^** | **2.61(1.86,3.65)^**^** | **3.70(2.64,5.20)^**^** | **2.89(1.47,5.68)^**^** | **3.74(1.82,7.69)^**^** | **2.25(1.17,4.33)^*^** | **2.67(1.34,5.32)^*^** |
| ≥ 6 | **2.68(2.16,3.33)^**^** | **5.82(4.56,7.42)^**^** | **3.04(2.18,4.24)^**^** | **5.08(3.61,7.15)^**^** | **2.43(1.20,4.91)^*^** | **3.80(1.81,7.97)^**^** | **3.15(1.90,5.20)^**^** | **4.39(2.67,7.19)^**^** |
| P for trend | **< 0.0001** | **< 0.0001** | **< 0.0001** | **< 0.0001** | 0.12 | **0.02** | **< 0.0001** | **< 0.0001** |

Footnotes: For all SDoH variables: Crude model was unadjusted. For each of eight dichotomized SDoH variables: Multivariable models refer to Model 2 which adjusted for age, sex, race, and other seven dichotomized SDoH variables. For cumulative number of unfavorable SDoH: Multivariable models refer to Model 1 which adjusted for age, sex, and race. Results of AOR (95% CI), p for trend and p-Value presented with bold valued were statistically significant with p < 0.05.

* p < 0.05, ** p < 0.001.

a Cumulative number of unfavorable SDoH was calculated by accumulating the eight dichotomized SDoH with a value of 0 for favorable level and a value of 1 for unfavorable level. Results of AORs (95% CI) for the cumulative number of unfavorable SDoH are based on Model 1, while AORs (95% CI) for eight individual sub-items of SDoH are based on Model 2.

Abbreviations: AOR, Adjusted odds ratio; CI, Confidence interval; PIR, Poverty-to-income ratio; SDoH, Social Determinants of Health.

**Supplementary Table S6 Association between Social Determinants of Health and cardiovascular disease in survey-weighted logistic regression models stratified by age groups, U.S. NHANES 2005-2018**

| Characteristic | Age 20 - 39 years | | Age 40 - 59 years | | Age ≥ 60 years | |
| --- | --- | --- | --- | --- | --- | --- |
| Variable | COR (95% CI) | AOR (95% CI) | COR (95% CI) | AOR (95% CI) | COR (95% CI) | AOR (95% CI) |
| Employment status |  |  |  |  |  |  |
| Employed, student, or retired | Reference | Reference | Reference | Reference | Reference | Reference |
| Unemployed | **3.25(2.27,4.66)^**^** | **2.37(1.59,3.54)^**^** | **3.74(3.16,4.43)^**^** | **2.33(1.88,2.88)^**^** | **1.69(1.45,1.98)^**^** | 1.53(1.29,1.82)**^**^** |
| Family income-to-poverty ratio |  |  |  |  |  |  |
| ≥ 300% | Reference | Reference | Reference | Reference | Reference | Reference |
| < 300% | **3.49(2.14,5.71)^**^** | **2.56(1.53,4.28)^**^** | **2.77(2.25,3.41)^**^** | **1.38(1.04,1.82)^*^** | **1.77(1.56,2.00)^**^** | **1.58(1.37,1.81)^**^** |
| Food security |  |  |  |  |  |  |
| Full security | Reference | Reference | Reference | Reference | Reference | Reference |
| Marginal, low, or very low security | **2.22(1.56,3.16)^**^** | 1.45(0.93,2.25) | **2.81(2.36,3.34)^**^** | **1.53(1.24,1.88)^**^** | **1.59(1.40,1.81)^**^** | **1.17(1.00,1.37)^*^** |
| Education level |  |  |  |  |  |  |
| High school graduate or higher | Reference | Reference | Reference | Reference | Reference | Reference |
| Less than high school | **1.73(1.19,2.51)^*^** | 1.15(0.78,1.70) | **1.79(1.46,2.19)^**^** | 1.13(0.91,1.40) | **1.49(1.32,1.69)^**^** | **1.22(1.05,1.42)^*^** |
| Covered by health insurance |  |  |  |  |  |  |
| Yes | Reference | Reference | Reference | Reference | Reference | Reference |
| No | 1.11(0.77,1.61) | **0.65(0.44,0.95)^*^** | 1.13(0.91,1.39) | **0.47(0.37,0.60)^**^** | 0.81(0.63,1.03) | **0.57(0.44,0.73)^**^** |
| Type of health insurance |  |  |  |  |  |  |
| Private | Reference | Reference | Reference | Reference | Reference | Reference |
| Government or none | **2.20(1.51,3.19)^**^** | 1.50(0.98,2.29) | **3.12(2.62,3.70)^**^** | **2.12(1.62,2.76)^*^** | **1.47(1.31,1.65)^*^** | **1.25(1.09,1.43)^*^** |
| Home ownership |  |  |  |  |  |  |
| Own home | Reference | Reference | Reference | Reference | Reference | Reference |
| Rent home or other arrangement | 1.42(0.92,2.21) | 1.12(1.02,1.28) | **2.09(1.76,2.47)^**^** | 1.19(0.97,1.48) | **1.55(1.37,1.75)^**^** | **1.21(1.05,1.39)^*^** |
| Marital status |  |  |  |  |  |  |
| Married or living with a partner | Reference | Reference | Reference | Reference | Reference | Reference |
| Not married nor living with a partner | 0.98(0.69,1.40) | 0.88(0.61,1.27) | **1.78(1.48,2.14)^**^** | 1.09(0.88,1.34) | **1.23(1.09,1.38)^**^** | **1.15(1.01,1.30)^*^** |
| Cumulative number of unfavorable SDoH^a^ |  |  |  |  |  |  |
| 0 | Reference | Reference | Reference | Reference | Reference | Reference |
| 1 | 0.64(0.23,1.81) | 0.66(0.23,1.85) | 1.32(0.89,1.96) | 1.37(0.92,2.04) | **1.64(1.32,2.04)^**^** | **1.78(1.43,2.22)^**^** |
| 2 | 0.77(0.30,1.97) | 0.81(0.31,2.09) | **2.30(1.50,3.52)^**^** | **2.42(1.59,3.69)^**^** | **1.84(1.47,2.31)^**^** | **2.11(1.67,2.65)^**^** |
| 3 | 1.07(0.44,2.61) | 1.14(0.46,2.82) | **2.53(1.76,3.63)^**^** | **2.73(1.89,3.94)^**^** | **1.91(1.51,2.42)^**^** | **2.28(1.78,2.92)^**^** |
| 4 | 2.32(1.09,4.94)**^*^** | **2.24(0.93,5.43)^*^** | **3.72(2.54,5.45)^**^** | **4.02(2.75,5.89)^**^** | **2.58(2.04,3.26)^**^** | **3.42(2.65,4.40)^**^** |
| 5 | **1.91(0.80,4.56)^*^** | **2.62(1.21,5.67)^*^** | **5.69(4.07,7.95)^**^** | **6.26(4.47,8.76)^**^** | **2.83(2.28,3.52)^**^** | **3.61(2.88,4.53)^**^** |
| ≥ 6 | **3.44(1.62,7.32)^**^** | **4.27(1.95,9.36)^**^** | **6.06(4.35,8.43)^**^** | **6.86(4.89,9.63)^**^** | **3.11(2.48,3.90)^**^** | **4.43(3.46,5.68)^**^** |
| P for trend | 0.23 | **0.002** | **< 0.0001** | **< 0.0001** | **< 0.0001** | **< 0.0001** |

Footnotes: For all SDoH variables: Crude model was unadjusted. For each of eight dichotomized SDoH variables: Multivariable models refer to Model 2 which adjusted for age, sex, race, and other seven dichotomized SDoH variables. For cumulative number of unfavorable SDoH: Multivariable models refer to Model 1 which adjusted for age, sex, and race. Results of AOR (95% CI), p for trend and p-Value presented with bold valued were statistically significant with p < 0.05.

* p < 0.05, ** p < 0.001.

a Cumulative number of unfavorable SDoH was calculated by accumulating the eight dichotomized SDoH with a value of 0 for favorable level and a value of 1 for unfavorable level. Results of AORs (95% CI) for the cumulative number of unfavorable SDoH are based on Model 1, while AORs (95% CI) for eight individual sub-items of SDoH are based on Model 2.

Abbreviations: AOR, Adjusted odds ratio; CI, Confidence interval; SDoH, Social Determinants of Health.

**Supplementary Table S7 Association between Social Determinants of Health and cardiovascular disease in survey-weighted logistic regression models using original categories, U.S. NHANES 2005-2018**

|  | Crude model |  | Model 1 |  | Model 2 |  |
| --- | --- | --- | --- | --- | --- | --- |
| SDoH Variables | COR (95% CI) | P - Value | AOR (95% CI) | P - Value | AOR (95% CI) | P - Value |
| Employment status |  |  |  |  |  |  |
| Employed, student, or retired | Reference |  | Reference |  | Reference |  |
| Unemployed | 1.80(1.64,1.98) | **<0.0001** | 3.00(2.69,3.36) | **<0.0001** | 2.25(1.98,2.55) | **< 0.001** |
| P for trend |  | **<0.0001** |  | **<0.0001** |  | **< 0.001** |
| Family income-to-poverty ratio |  |  |  |  |  |  |
| > 500% | Reference |  | Reference |  | Reference |  |
| 300% - 500% | 1.25(1.06,1.48) | **0.01** | 1.29(1.09,1.54) | **0.004** | 1.14(0.95,1.37) | 0.15 |
| 100% - 300% | 2.12(1.84,2.44) | **<0.0001** | 2.07(1.78,2.40) | **<0.0001** | 1.43(1.21,1.68) | **< 0.0001** |
| <100% | 1.96(1.66,2.31) | **<0.0001** | 3.17(2.68,3.76) | **<0.0001** | 1.44(1.15,1.78) | **0.002** |
| P for trend |  | **0.023** |  | **<0.0001** |  | **<0.0001** |
| Food security |  |  |  |  |  |  |
| Full security | Reference |  | Reference |  | Reference |  |
| Marginal security | 1.00(0.86,1.15) | 0.97 | 1.75(1.52,2.03) | **<0.0001** | 1.23(1.06,1.44) | **0.01** |
| Low security | 1.32(1.16,1.51) | **<0.0001** | 2.58(2.22,3.00) | **<0.0001** | 1.59(1.35,1.87) | **<0.0001** |
| Very low security | 1.52(1.27,1.81) | **<0.0001** | 3.29(2.64,4.11) | **<0.0001** | 1.78(1.42,2.23) | **<0.0001** |
| P for trend |  | **<0.0001** |  | **<0.0001** |  | **<0.0001** |
| Education level |  |  |  |  |  |  |
| College | Reference |  | Reference |  | Reference |  |
| Some college or AA degree | 1.45(1.26,1.66) | **<0.0001** | 1.61(1.40,1.84) | **<0.0001** | 1.28(1.11,1.48) | **<0.001** |
| High school | 1.89(1.65,2.18) | **<0.0001** | 1.75(1.52,2.03) | **<0.0001** | 1.24(1.05,1.48) | **0.002** |
| Less than high school | 2.48(2.15,2.87) | **<0.0001** | 2.14(1.82,2.52) | **<0.0001** | 1.29(1.10,1.51) | **0.01** |
| P for trend |  | **<0.0001** |  | **<0.0001** |  | **0.01** |
| Covered by health insurance |  |  |  |  |  |  |
| Yes | Reference |  | Reference |  | Reference |  |
| No | 0.45(0.39,0.52) | **<0.0001** | 1.20(1.03,1.39) | **0.02** | 0.69(0.59,0.81) | **<0.0001** |
| P for trend |  | **<0.0001** |  | **0.012** |  | **<0.0001** |
| Type of health insurance |  |  |  |  |  |  |
| Private | Reference |  | Reference |  | Reference |  |
| Government | 0.35(0.33,0.39) | **<0.0001** | 0.49(0.45,0.54) | **<0.0001** | 1.16(1.06,1.28) | **0.001** |
| No insurance | 0.23(0.20,0.26) | **<0.0001** | 0.74(0.64,0.86) | **<0.001** | 0.84(0.73,1.18) | 0.27 |
| P for trend |  | **<0.0001** |  | **<0.001** |  | 0.212 |
| Home ownership |  |  |  |  |  |  |
| Own home | Reference |  | Reference |  | Reference |  |
| Rent home | 0.81(0.73,0.89) | **<0.0001** | 1.81(1.63,2.01) | **<0.0001** | 1.27(1.12,1.43) | **<0.001** |
| Other arrangement | 0.99(0.74,1.32) | 0.94 | 1.35(0.98,1.85) | 0.06 | 1.02(0.73,1.42) | 0.93 |
| P for trend |  | **<0.001** |  | **0.032** |  | **0.041** |
| Marital status |  |  |  |  |  |  |
| Married or living with a partner | Reference |  | Reference |  | Reference |  |
| Never married | 0.40(0.33,0.48) | **<0.0001** | 1.19(0.95,1.47) | 0.13 | 0.85(0.68,1.05) | **0.13** |
| Widowed | 4.30(3.81,4.85) | **<0.0001** | 1.30(1.14,1.48) | **<0.001** | 1.06(0.93,1.20) | **0.40** |
| Divorced | 1.41(1.23,1.63) | **<0.0001** | 1.27(1.08,1.48) | **0.003** | 0.93(0.78,1.10) | 0.37 |
| Separated | 1.24(0.98,1.57) | 0.07 | 1.71(1.29,2.26) | **<0.001** | 1.10(0.83,1.45) | 0.52 |
| P for trend |  | 0.452 |  | **<0.001** |  | 0.362 |

Footnotes: Original categories for each of SDoH variables have shown in Table S1. Reference was set based on favorable SDoH groups. For the eight sub-items of SDoH: Crude model was unadjusted; Model 1 adjusted for age, gender, and race/ethnicity; Model 2 adjusted for age, gender, race/ethnicity, and the other seven sub-items of SDoH. Results of COR (95% CI), AOR (95% CI), p for trend and p-Value presented with bold valued were statistically significant with p < 0.05.

Abbreviations: AA, Associate's Degree; AOR, Adjusted odds ratio; CI, Confidence interval; COR, Crude odds ratio; SDoH, Social Determinants of Health.

**Supplementary Table S8 Association between five domains about Social Determinants of Health and cardiovascular disease in survey-weighted logistic regression models, U.S. NHANES 2005-2018**

|  | Crude model | | Model 1 | | Model 2 | |
| --- | --- | --- | --- | --- | --- | --- |
| SDoH Variables | COR (95% CI) | P- Value | AOR (95% CI) | P - Value | AOR (95% CI) | P - Value |
| **Economic Stability** |  |  |  |  |  |  |
| 0 | Reference |  | Reference |  | Reference |  |
| 1 | 1.49(1.32,1.69) | **<0.0001** | 1.35(1.18,1.53) | **<0.0001** | 1.28(1.12,1.45) | **< 0.0001** |
| 2 | 1.55(1.38,1.75) | **<0.0001** | 2.70(2.38,3.06) | **<0.0001** | 2.38(2.05,2.76) | **< 0.0001** |
| 3 | 3.01(2.63,3.45) | **<0.0001** | 6.39(5.46,7.48) | **<0.0001** | 5.11(4.30,6.08) | **< 0.0001** |
| P for trend |  | **<0.0001** |  | **<0.0001** |  | **< 0.0001** |
| **Education Access and Quality** |  |  |  |  |  |  |
| 0 | Reference |  | Reference |  | Reference |  |
| 1 | 1.76(1.59,1.96) | **<0.0001** | 1.49(1.31,1.68) | **<0.0001** | 1.04(0.91,1.18) | **0.01** |
| P for trend |  | **<0.0001** |  | **<0.0001** |  | **0.01** |
| **Health Care Access and Quality** |  |  |  |  |  |  |
| 0 | Reference |  | Reference |  | Reference |  |
| 1 | 2.82(2.59,3.07) | **<0.0001** | 2.03(1.84,2.23) | **<0.0001** | 1.38(1.23,1.54) | **< 0.0001** |
| 2 | 1.15(0.97,1.54) | **<0.0001** | 1.50(1.29,1.75) | **<0.001** | 0.96(0.80,1.15) | 0.66 |
| P for trend |  | 0.678 |  | **<0.0001** |  | 0.252 |
| **Neighborhood and Built Environment** |  |  |  |  |  |  |
| 0 | Reference |  | Reference |  | Reference |  |
| 1 | 1.08(1.08,1.08) | **<0.0001** | 1.76(1.60,1.95) | **<0.0001** | 1.21(1.08,1.36) | **0.001** |
| P for trend |  | **<0.0001** |  | **<0.0001** |  | **0.001** |
| **Social and Community Context** |  |  |  |  |  |  |
| 0 | Reference |  | Reference |  | Reference |  |
| 1 | 1.24(1.13,1.35) | **<0.0001** | 1.28(1.16,1.42) | **<0.0001** | 0.96(0.86,1.07) | 0.49 |
| P for trend |  | **<0.0001** |  | **<0.001** |  | 0.371 |

Footnotes: There are five domains of SDoH framework: economic stability (employment status, family income-to-poverty ratio, food security), education access and quality (education level), health care access and quality (health insurance coverage, type of health insurance), neighborhood and built environment (home ownership), and social and community context (marital status). For cumulative number of unfavorable SDoH from each domain: Crude model was unadjusted; Model 1 adjusted for age, sex, and race; Model 2 adjusted for age, sex, race, and the other sub-items of SDoH from the other domains. Results of COR (95% CI), AOR (95% CI), p for trend and p-Value presented with bold valued were statistically significant with p < 0.05.

^a^ Cumulative number of unfavorable SDoH from five domains was calculated by accumulating the dichotomized SDoH with a value of 0 for favorable level and a value of 1 for unfavorable level from each domain.

Abbreviations: AOR, Adjusted odds ratio; CI, Confidence interval; COR, Crude odds ratio; SDoH, Social Determinants of Health.

**Supplementary Table S9 Further adjustments in sensitivity analyses for Social Determinants of Health associated with cardiovascular disease by survey-weighted logistic regression models, U.S. NHANES 2005-2018**

| **SDoH Variables** | **Adjusted for survey waves, AOR (95% CI)** | **Adjusted for BMI, smoking, dringking, and recreational activity, AOR (95% CI)** | **Adjusted for hypertension, diabete, creatinine, HDL, and TC, AOR (95% CI)** | **Adjusted for BMI, smoking, dringking, recreational activity, hypertension, diabete, creatinine, HDL, and TC, AOR (95% CI)** |
| --- | --- | --- | --- | --- |
| Employment status |  |  |  |  |
| Employed, student, or retired | Reference | Reference | Reference | Reference |
| Unemployed | **2.27(2.00,2.57)**^**^ | **1.98(1.73,2.26)**^**^ | **2.15(1.87,2.47)^**^** | **1.90(1.64,2.19)^**^** |
| Family income-to-poverty ratio |  |  |  |  |
| ≥ 300% | Reference | Reference | Reference | Reference |
| < 300% | **1.40(1.24,1.57)**^**^ | **1.28(1.13,1.46)^**^** | **1.31(1.15,1.48)^**^** | **1.27(1.11,1.45)^**^** |
| Food security |  |  |  |  |
| Full security | Reference | Reference | Reference | Reference |
| Marginal, low, or very low security | **1.54(1.36,1.73)^**^** | **1.38(1.21,1.57)^**^** | **1.41(1.24,1.60)^**^** | **1.34(1.17,1.53)^**^** |
| Education level |  |  |  |  |
| High school graduate or higher | Reference | Reference | Reference | Reference |
| Less than high school | 1.02(0.90,1.16) | 0.95(0.82,1.10) | 0.99(0.85,1.14) | 1.17(0.96,1.40) |
| Covered by health insurance |  |  |  |  |
| Yes | Reference | Reference | Reference | Reference |
| No | **0.69(0.59,0.81)^**^** | **0.71(0.59,0.85)^*^** | **0.84(0.70,0.99)^*^** | **0.80(0.66,0.97)^*^** |
| Type of health insurance |  |  |  |  |
| Private | Reference | Reference | Reference | Reference |
| Government or none | **1.38(1.24,1.55)^**^** | **1.39(1.23,1.58)^**^** | **1.33(1.18,1.50)^**^** | **1.35(1.18,1.54)^**^** |
| Home ownership |  |  |  |  |
| Own home | Reference | Reference | Reference | Reference |
| Rent home or other arrangement | **1.26(1.12,1.41)^**^** | **1.25(1.10,1.41)^**^** | **1.27(1.12,1.43)^**^** | **1.25(1.09,1.42)^*^** |
| Marital status |  |  |  |  |
| Married or living with a partner | Reference | Reference | Reference | Reference |
| Not married nor living with a partner | 0.97(0.87,1.08) | **1.11(1.02,1.26)^*^** | **1.12(1.01,1.24)^*^** | **1.06(0.94,1.18)^*^** |
| Cumulative number of unfavorable SDoH^a^ |  |  |  |  |
| 0 | Reference | Reference | Reference | Reference |
| 1 | **1.45(1.21,1.74)^**^** | **1.25(1.04,1.51)^*^** | **1.40(1.15,1.70)^**^** | **1.23(1.02,1.49)^*^** |
| 2 | **1.70(1.40,2.08)^**^** | **1.45(1.19,1.76)^**^** | **1.59(1.28,1.97)^**^** | **1.41(1.15,1.72)^**^** |
| 3 | **1.86(1.55,2.24)^**^** | **1.51(1.23,1.85)^**^** | **1.68(1.37,2.06)^**^** | **1.45(1.17,1.80)^**^** |
| 4 | **3.17(2.62,3.85)^**^** | **2.53(2.04,3.13)^**^** | **2.95(2.39,3.65)^**^** | **2.51(2.01,3.14)^**^** |
| 5 | **3.94(3.24,4.79)^**^** | **2.80(2.26,3.45)^**^** | **3.43(2.76,4.26)^**^** | **2.69(2.16,3.35)^**^** |
| ≥ 6 | **5.35(4.44,6.43)^**^** | **3.64(2.98,4.44)^**^** | **4.61(3.79,5.60)^**^** | **3.35(2.75,4.09)^**^** |
| P for trend | **< 0.0001** | **< 0.0001** | **< 0.0001** | **< 0.0001** |

Footnotes: For each of eight dichotomized SDoH variables: Sensitivity analyses were performed based on Model 2, and plus adjusted by adding variables including survey waves, BMI, smoking, dringking, recreational activity, hypertension, diabete, creatinine, HDL, and TC, respectively. For cumulative number of unfavorable SDoH: Multivariable models were based on Model 1 and plus adjusted other variables. Results of AOR (95% CI), p for trend and p-Value presented with bold valued were statistically significant with p < 0.05.

* p < 0.05, ** p < 0.001.

^a^ Cumulative number of unfavorable SDoH was calculated by accumulating the eight dichotomized SDoH with a value of 0 for favorable level and a value of 1 for unfavorable level. Results of AORs (95% CI) for the cumulative number of unfavorable SDoH are based on Model 1, while AORs (95% CI) for eight individual sub-items of SDoH are based on Model 2.

Abbreviations: AOR, Adjusted odds ratio; CI, Confidence interval; BMI, Body mass index; HDL, high-density lipoprotein; TC, [total cholesterol](https://www.sciencedirect.com/topics/biochemistry-genetics-and-molecular-biology/cholesterol-blood-level); SDoH, Social Determinants of Health.
